# The verification of wildland–urban interface fire evacuation models

**DOI:** 10.1007/s11069-023-05913-2

**Published:** 2023-03-28

**Authors:** E. Ronchi, J. Wahlqvist, A. Ardinge, A. Rohaert, S. M. V. Gwynne, G. Rein, H. Mitchell, N. Kalogeropoulos, M. Kinateder, N. Bénichou, E. Kuligowski, A. Kimball

**Affiliations:** 1 Department of Fire Safety Engineering, Lund University, Lund, Sweden; 2 Movement Strategies, London, UK; 3 Imperial College London, London, UK; 4 National Research Council, Ottawa, Canada; 5 Royal Melbourne Institute of Technology, Melbourne, Australia; 6 Fire Protection Research Foundation, Quincy, USA

**Keywords:** Wildfire, Evacuation, Egress, Verification, Simulation, Fire

## Abstract

This paper introduces a protocol for the verification of multi-physics wildfire evacuation models, including a set of tests used to ensure that the conceptual modelling representation of each modelling layer is accurately implemented, as well as the interactions between different modelling layers and sub-models (wildfire spread, pedestrian movement, traffic evacuation, and trigger buffers). This work presents a total of 24 verification tests, including (1) 4 tests related to pedestrians, (2) 15 tests for traffic evacuation, (3) 5 tests concerning the interaction between different modelling layers, along with 5 tests for wildfire spread and trigger buffers. The evacuation tests are organized in accordance with different core components related to evacuation modelling, namely Population, Pre-evacuation, Movement, Route/destination selection, Flow constraints, Events, Wildfire spread and Trigger buffers. A reporting template has also been developed to facilitate the application of the verification testing protocol. An example application of the testing protocol has been performed using an open wildfire evacuation modelling platform called WUI-NITY and its associated trigger buffer model k-PERIL. The verification testing protocol is deemed to improve the credibility of wildfire evacuation model results and stimulate future modelling efforts in this domain.

**Supplementary Information:**

The online version contains supplementary material available at 10.1007/s11069-023-05913-2.

## Introduction

Fires in the wildland–urban interface (WUI) are associated with severe negative consequences, such as large community evacuations, property losses, disruption to social processes, injuries, and even fatalities (Haynes et al. 2019; Kuligowski 2020). This issue has led to significant research developments in the wildfire evacuation domain (Haghani et al. 2022). Seminal research in the domain of hurricane disasters (Whitehead 2003) has provided estimates of costs of evacuation by breaking them down into loss of income, transportation costs/time, direct costs while away (e.g., food, lodging, entertainment). Estimates of wildfire evacuation costs were obtained considering hurricane evacuation costs together with estimates of number of evacuees and length of evacuation (Thomas et al. 2017). This resulted in an estimate of US$ 3 billion in costs due only to evacuation in the US.

Given their scale and complexity, it is contended that wildfire incidents require a multi-disciplinary approach to assess their impact on a community and the effectiveness of subsequent mitigation efforts. The importance of wildfire evacuation is highlighted by the consequences that such scenarios may have on communities and how the possible sequence of events are associated with evacuation issues (e.g., availability of evacuation modes, number of evacuees in relation to the fire, organisational response needed, etc.) as presented in a report investigating a worldwide list of wildfire evacuation (Ronchi et al. 2021b). In this context, evacuation models are a useful tool for assessing the impact of a wildfire on a community and identifying means to reduce their negative consequences (Ronchi and Gwynne 2019). Simulation models are increasingly used to guide the development of evacuation plans for WUI communities and aid real-time emergency management. However, many limitations exist among current models (Kuligowski 2020). For example, many existing wildfire evacuation models have not focused on policy responses or testing of different scenarios, with only a few exceptions (Chen et al. 2020; Zhao and Wong 2021). In particular, their predictive capabilities have not been systematically tested yet (Ronchi et al. 2020). Past research efforts (Cova et al. 2005; Dennison et al. 2007; Beloglazov et al. 2016; Veeraswamy et al. 2018; Li et al. 2019; Wahlqvist et al. 2021a) aimed at representing the three modelling layers (and their interactions) involved in a wildfire evacuation event, namely (1) fire spread, (2) human response (including the representation of different behaviours generally through a distribution of evacuation responses) and movement to transport systems, and (3) traffic movement to safety. While present and projected conditions across these core components are represented in a number of ways in currently available models, they lead to models with a significant level of complexity and involve sophisticated interactions among the simulation domains yet (Ronchi et al. 2020).

In this context, the definition of a verification and validation (V&V) protocol is a necessary tool for testing existing and future wildfire evacuation models. Verification is here defined as the process of determining that a correct implementation of the developer's conceptual description has been performed. Validation refers to the process of determining the degree to which a simulation is an accurate representation of the real world. The importance of verification and validation for model users has been highlighted in a recent survey conducted in the crowd evacuation modelling domain (Lovreglio et al. 2020). Survey results showed indeed that V&V is considered by model users the most important factor when selecting/using a model. While validation efforts rely on the availability of data—which unfortunately are currently very scarce and relate to only a limited set of aspects of wildfire evacuation scenarios (Zhao et al. 2022; Rohaert et al. 2023; Wong et al. 2022; Katzilieris et al. 2022a)—a verification protocol is an achievable basic first step towards improving model credibility. It is therefore noted that this paper focusses on verification, as validation would require a wide range of wildfire evacuation data to be available to develop a comprehensive V&V protocol.

Existing research concerning the verification of traffic evacuation models is not specifically addressing the wildfire evacuation domain. In fact, current tests and procedure are generally designed for traffic models for general use (Rakha et al. 1996), focussing on both analytical/macroscopic (Kaczmarek and Sac 2016; Chalfen and Kamińska 2018) and microscopic approaches (Ciuffo and Punzo 2010; Tian et al. 2015). Such research often specifically addresses certain types of models (or sub-models) or application domains (Namekawa et al. 2007; Chalfen and Kamińska 2018; Yu et al. 2021), thus impeding their applicability to a variety of modelling tools and scenarios. General guidelines on the verification/calibration that can be applied to traffic evacuation models exist (Whitner and Balci 1989; Rakha et al. 1996; Hellinga 1998), but given its broad nature, it would require dedicated efforts to be concretely applied to wildfire evacuation models. In addition, these documents do not consider explicitly the interaction with other modelling layers which are typically present in wildfire evacuation models (e.g., pedestrian and traffic models). This limits the scope of application in this domain and in turn potentially affects the user trust over model results. In other words, the lack of dedicated literature and guidelines on wildfire evacuation model verification is likely hindering their development and use.

Inspiration for V&V of wildfire evacuation models can be borrowed from the domain of crowd evacuation models where documents and guidelines exist (Ronchi et al. 2013; Rimea 2016; International Standards Organization 2020). These documents describe how verification can be performed in several ways, among which a common approach is to employ a set of hypothetical test cases which ensure that the key features and components of a modelling tool are implemented correctly. An additional issue to consider specifically for wildfire evacuation multi-layer models is to establish that that individual modelling layers in isolation and the interactions between modelling layers work as intended.

To the best of our knowledge, no verification protocol is currently available in the wildfire evacuation domain. To address this issue, an international project (Ronchi et al. 2021a) was initiated with the goal of defining a verification protocol that could be applied to existing and future wildfire evacuation models. The project team involved a range of expertise in the three core layers of wildfire evacuation modelling (fire, pedestrian and traffic) and had specific experience in the development of standard V&V protocols (e.g., in the crowd evacuation modelling domain (International Standards Organization 2020)). This paper presents the key results of this research effort, as it introduces the workflow employed for the definition of the verification tests, and it presents them along with an example application to the freely available multi-layer platform called WUI-NITY (Wahlqvist et al. 2021a). Given the type of modelling under consideration, this work is intended primarily for fire engineers/managers using wildfire evacuation models rather than transportation engineers/planners. The model WUI-NITY was chosen here since it is a modelling platform which is deliberately designed to couple different modelling layers for the specific application of wildfire evacuation modelling. The platform is intended as model-agnostic, so that in the future could be linked with other models in the key domains (fire, pedestrian and traffic) of wildfire evacuation modelling. This is deemed to stimulate further research in this domain and facilitate future applications of the protocol to existing and future wildfire evacuation models. The terminology in use to present the verification protocol is deliberately in line with the one in use in the fire safety to facilitate its future use in this domain.

## The verification testing workflow

According to Oreskes et al. (1994), verification intends “to say that [the model’s] truth has been demonstrated, which implies its reliability as a basis for decision making”. In the modelling context, verification is generally intended as the process of ensuring that a conceptual model has been correctly implemented.

Model verification has been undertaken in several other domains with the aim to improve the credibility of models and in turn increase the pool of users. A known example is in the domain of pedestrian simulations where a testing protocol (International Standards Organization 2020) has been adopted by several evacuation model developers, after being demanded by this modelling community for several years (Ronchi 2014).

**Fig. 1:**
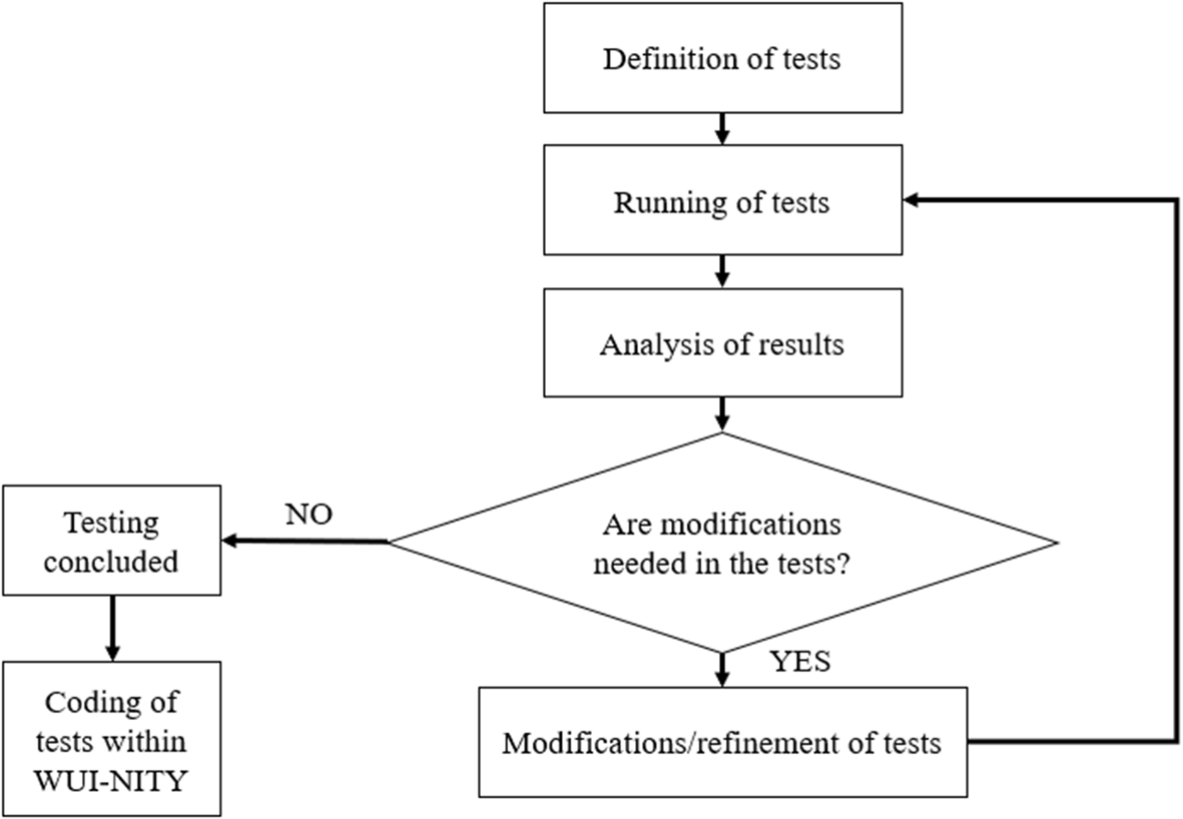
Iterative loop of workflow for verification testing

The verification testing workflow has been performed following an iterative loop. In this loop (see Fig. 1), the first step was to define the key variables to test along with the associated verification tests the software should go through. This was broadly based on a corresponding existing document in the crowd modelling domain, i.e., ISO 20414 (International Standards Organization 2020) and it was complemented with the variables identified in a previous literature review on the use of traffic models for WUI fire evacuations (Intini et al. 2019). The latter is a review which can be used to identify how traffic model can be used for WUI fire evacuation scenarios. Each test included defining an objective, drawing geometrical boundaries, representing a scenario, and documenting expected results, the method used and the actions the user needs to take. After the tests were defined, they were preliminarily applied to the simulation software WUI-NITY (both evacuation and wildfire sub models) and its associated trigger buffer model k-PERIL. Trigger buffers indicate a point or a perimeter ahead of a community that when reached by a wildfire should inform a safe evacuation order being given (Mitchell et al. 2023). The results produced were compared to expected hand calculated results and the differences were analysed. Through the analysis of the results, improvements to the tests were made with the goal to further scrutinize model capabilities, e.g., additions of sub-cases, modification in geometries, additional scenarios, and new tests with interactions to modelling layers. Once improvements in the tests were made, they were run again, and the new results were analysed. Then, the iterative loop continued until a stable set of tests was identified. The verification tests were then run automatically through the source code of WUI-NITY so that possibly newer versions of the code could be automatically tested. This was deemed a suitable approach for such type of verification testing.

## Categorization of tests

The verification tests were constructed following the structure of the ISO 20414 standard (International Standards Organization 2020). Verification tests therefore consisted of six parts:

1. *Objective* description of what component or behaviour is being tested and what model/method it is being compared against to ensure the parameter is functioning properly,
2. *Geometry* the configuration of the test,
3. *Scenario(s)* the evacuation scenario(s) to be simulated,
4. *Expected result* the result (qualitative or quantitative) that the evacuation model should produce
5. *Test method* the qualitative (e.g., visualisation of the represented behaviour) or quantitative—e.g., comparison of evacuation time estimates (often referred as evacuation times in fire safety engineering), flows, etc.—method employed for the comparison between the expected result and the simulation results, and
6. *User’s actions* the actions required of the tester while performing and presenting the tests (Ronchi et al. 2013, 2014, 2020).

When presenting the suggested tests for the verification of evacuation models, they were organized according to the elements which are considered necessary to meet the most basic representation of a scenario. These core components of evacuation models were defined based on previous existing categorization which were adapted for the specific domain under consideration (Gwynne et al. 2015):

1. *Population* intended here as physical characteristics of pedestrians and their interaction with vehicles (number of vehicles available, and their initial distance)
2. *Pre-evacuation* time to respond to the event and their distribution in the domain
3. *Movement* variables related to movement on the road
4. *Route/destination selection* variables related to route and destination choice
5. *Flow constraints* variables related to vehicles flows
6. *Events* events affecting flow as a result of policy response/decisions
7. *Wildfire spread* variables related to wildfire propagation
8. *Trigger buffers* variables related to the trigger buffers

Verification tests were defined as hypothetical ideal tests that are designed to analyse the main features of evacuation models.

## Key factors selected for verification testing

Table 1 presents the suggested factors for consideration in the verification testing of WUI evacuation. In order to keep the number of tests to a manageable size in this first verification protocol, the tests primarily address the traffic evacuation component and its interaction with other modelling layers. This choice was made because the temporal scale of evacuation time is driven in most cases by this modelling domain (Ronchi et al. 2020).

**Table 1:** Key factors for verification of wildfire evacuation and associated core component

Category	Variable
*Pre-evacuation*	Pedestrian re-distribution
	Response curve
*Movement*	Uni-directional single vehicle flow (one road type)
	Uni-directional single vehicle flow (multiple road types)
	Background traffic
	Speed–density and flow–density relationships
	Group evacuation
	Lane changing/overtaking
	Acceleration/deceleration
	Type of intersection
	Vehicle demand versus arrival distribution
*Route/destination selection*	Forced destination
	Destination choice in traffic
	Route choice in traffic
*Flow constraints*	Change in carriageway configuration
	Flow at destination
*Population*	Max vehicles per household
	Pedestrian walking speed
	Pedestrian distance to vehicle
*Events*	Vehicle speed reduction in reduced visibility conditions
	Road crash
	Route loss
	Lane reversal
	Loss of shelter or exit
	Refuge capacity

The key selected model features/components examined are here described along with how they are defined, and what is being tested. Many of the features are equivalent to those included in V&V testing for fire evacuation in buildings and ships (Ronchi et al. 2013, 2014; International Maritime Organization 2016; Rimea 2016; International Standards Organization 2020), but rather than considering people and buildings/ships, the factors are modified to be applicable for vehicles and road networks. In addition, since there is no currently existing standardized verification testing protocol for evacuation models used in the WUI context, suggestions for new features/components to test are included concerning pedestrian and traffic layers and interactions between those layers and the wildfire layer.

This testing protocol also includes a dedicated set of simplified verification tests representing wildfire scenarios of increasing complexity. These tests can be used to both verify the proper integration of a wildfire model into the multi-layer modelling framework (e.g., FARSITE (Finney et al. 1998) into WUI-NITY), as well as the testing of trigger buffer models (see Table 2).

**Table 2:** Wildfire scenarios and variables under consideration for the verification of both the wildfire spread model and trigger buffer models coupled with wildfire evacuation models

Type of scenarios	Variables under consideration
Homogenous	Base case with flat land, uniform fuel type and zero wind
Varying Fuel	Fuel types
Set wind	Wind
Valley	Topography
Heterogeneous	Fuel type, wind, topography

The following description presented gives an overview of the factors/components taken into consideration. Each test was given a code in which there are letters representing the modelling layer under scrutiny (W for wildfires, P for pedestrians, T for traffic, B for trigger buffer) and a number. The readers are directed to the supplementary material of this paper and the full report associated with this document for a detailed description of each test (Ronchi et al. 2021a).

### Test P.1: pedestrian re-distribution

The spatial map used to perform evacuation simulation contains a traffic network and households. This test aims at verifying that households can connect to the traffic network through a traffic node and investigate how the software addresses the case in which households are not directly connected to the road network.

### Test P.2: maximum number of available vehicles per household

The number of vehicles to which a household has access and uses in an evacuation varies. The more vehicles used, the more capacity there is for additional evacuees, belongings, and supplies. The assumptions of number of vehicles available at each household should be tested with a probability to take additional vehicles. This test ensures that the number of vehicles entering the traffic model correspond to the implemented numbers of vehicles assigned to each household and that the number of vehicles on the road does not exceed the population number (e.g., each vehicle needs at least one driver).

### Test P.3: response curve

The response curve represents the time it takes for people to pick up on cues from an approaching wildfire and start evacuating. Cues can be smoke from wildfire, neighbourhood activity, radio messages, evacuation warnings, etc. (McLennan et al. 2012, 2019; Ronchi et al. 2017; Sutton and Kuligowski 2019). People can start evacuating before evacuation orders are issued, delay their evacuation, or not evacuate at all (Kuligowski 2020).

### Test P.4: pedestrian walking speed

The pedestrian walking speed checks that a pedestrian can walk from their starting location to a destination to either reach safety or a traffic node to evacuate the area with an assigned walking speed. The walking speed may vary between people in relation to their physical abilities (Gwynne et al. 2016).

### Test PT.1: pedestrian distance to vehicle

The pedestrian walking distance variable investigates whether pedestrians can walk from their starting location to a destination to either reach safety or a traffic node to evacuate the area using their vehicle. The walking distance varies between pedestrians, i.e., some are further away from the access to the traffic system or must move around obstacles and adopt a higher walking distance than others.

### Test T.1: uni-directional single vehicle flow

Each road type has a corresponding speed limit which can be adopted by vehicles driving at free-flow speed. The free-flow speed should change if the vehicle enters another road type with a different speed limit.

### Test T.2: background traffic

The background traffic represents vehicles present on a road which are not actively evacuating (Intini et al. 2019). This type of traffic can affect the speed for all other vehicles since the road becomes more occupied and slower driving behaviour is adapted to the new traffic conditions.

### Test T.3: change in carriageway configuration

Change in carriageway configuration refers to a change in the type of cross section. For example, this includes the case of two consecutive roads with different number of lanes, widths, etc.

### Test T.4: speed–density and flow–density relationships

This test evaluates the relationships used to relate speed–density and flow–density (Dixit and Wolshon 2014; Rohaert et al. 2023) and how an increase in traffic density changes the speed and flow of the traffic. The speed is generally reduced with increases in density, while the flow increases with increasing density to a point where maximum flow (capacity) is reached; at which point, the flow reduces with increasing density.

### Test T.5: vehicle speed reduction in reduced visibility conditions

Traffic during wildfire evacuation can encounter smoke which can obscure the drivers’ views and make it unsafe to drive at the usual speeds for that specific road type. This causes the adopted speeds to decrease with decreasing visibility conditions, in addition to the potential speed reduction already adopted for increasing traffic densities (Wetterberg et al. 2021; Intini et al. 2022).

### Test T.6: flow at destination

The area where an evacuation is taking place usually has destinations or exits for leaving the area where evacuees can reach safety. The flows at these destinations should not exceed a maximum arrival flow rate and the remaining traffic density is reduced as speed and flow increases.

### Test T.7: group evacuation

Group evacuation with friends and family can occur if the members of a household (or more than one household) take one or multiple vehicles with the goal of evacuating close together on the roadways (Lovreglio et al. 2019). Multiple vehicles in close proximity within the traffic flow can be difficult to model with the presence of surrounding traffic, but the vehicles should be leaving the household at about same time.

### Test T.8: lane changing/overtaking

The speed at which people drive at for specific road types varies between drivers. People drive differently because they have different vehicles, driving experience, personal behaviours, etc. (Gray and Regan 2005). This difference in speed can cause the faster vehicles to change lanes and overtake to keep their current driving speed.

### Test T.9: acceleration/deceleration

Acceleration and deceleration of individual vehicles occur during evacuation in response to surrounding traffic or approaching intersections or roundabouts (Zhi et al. 2010). Vehicles can also alter their speed due to other reasons, such as encountering a slope (Martens et al. 1997).

### Test T.10: road crash

Road crashes can occur during evacuations—especially when environmental conditions deteriorate (Collins et al. 2014). With the increase in traffic density and an ongoing emergency, a higher number of vehicles are at risk of causing, engaging in or encountering a road crash (Dixit and Wolshon 2014). A road crash could cause certain lanes to be blocked or stop the traffic flow completely.

### Test T.11: intersection

Intersections where two or more roads meet can be signalised or unsignalised in relation to how designers manage the traffic evacuation flow (Parr et al. 2016). Other types of intersections can include roundabouts which can handle higher flows, and on- and off-ramps to highways, where the flows merge or disperse.

### Test T.12: forced destination

During evacuation, the destination chosen may not necessarily be the closest or one with the shortest journey time. Movement to a destination can also be linked to a set of activities or events such as evacuees picking up family members, avoiding road closures, and choosing a destination that is more familiar or offers higher levels of comfort (Murray-Tuite and Mahmassani 2003; Akbarzadeh and Wilmot 2015). A forced destination may be used to represent the case of choosing (either voluntary or due to a mandatory order) a destination to avoid the fire threat. Forced destinations, in this case, are used to override a given destination choice.

### Test T.13: destination choice in traffic

Destination choice refers to the evacuees’ decision on what destination to travel to reach safety during a wildfire (Wong et al. 2020, 2022). The decision can be based on the shortest, fastest, or a specified route to a destination, and their availability. Other conditions may also affect this decision, such as the presence of smoke (Wetterberg et al. 2021) or the choice of familiar routes by evacuees (Akbarzadeh and Wilmot 2015).

### Test T.14: route choice in traffic

Route choice in traffic refers to the evacuee’s decision on which route to take to reach a destination (Wong et al. 2020). This decision is tested separately from destination choice and can depend on the time or distance to traverse the route (faster or shorter) but can also be affected by other conditions such as route familiarity (Colonna et al. 2016), or the smoke/fire conditions blocking or impeding a route (Wetterberg et al. 2021). In other words, the traffic modeller should be able to consider the case in which the shortest route does not correspond to the fastest route (due to congestion).

### Test T.15: vehicle demand versus arrival distribution

Vehicle demand versus arrival distribution refers to the number of vehicles that leave the starting point of the road network and the number that arrive at the destination within the road network. In principle, the number of vehicles starting the evacuation should be the same as the number of vehicles that reach the destination and successfully evacuate unless the model assumes vehicles can break down or stop along the way.

### Test WT.1: route loss

Route loss refers to an event during which a road section is no longer available for use during evacuation. When a route is closed, other routes need to be used to allow the remaining traffic to evacuate. A route may be closed due to its the proximity to the fire front or smoke, due to a crash, due to downed trees or hazardous power lines.

### Test WT.2: lane reversal

Lane reversal (contraflow) is an option that can be implemented on roads with larger capacity (i.e., a higher number of lanes) by officials to increase the number of lanes open in the direction of evacuation (Wolshon 2001). This also means that the number of lanes for emergency personnel are reduced. The increase of capacity offered by lane reversal helps to increase traffic flow and reduce evacuation times (Akbarzadeh and Wilmot 2015). Considering contraflow a policy decision, it may be argued that this feature is not inherent in an evacuation model. Nevertheless, it was decided to have a test concerning this feature as it may be implemented into a wildfire evacuation model as a consequence of wildfire spread.

### Test WT.3: loss of exit or shelter

Loss of exit or shelter refers to when a destination of the evacuating traffic is no longer available for use. Reasons for exit/shelter closure can include cases where the wildfire is blocking access to the destination and the remaining traffic needs to be redirected to another destination.

### Test WT.4: refuge capacity

Refuge capacity refers to the number of people allowed within an emergency shelter. When a shelter has reached maximum capacity, any additional newcomers are required to find another shelter (Blanchi et al. 2018). The notification of a shelter being full can be announced by different means (Sutton and Kuligowski 2019).

### Test B.1: homogenous

**Fig. 2:**
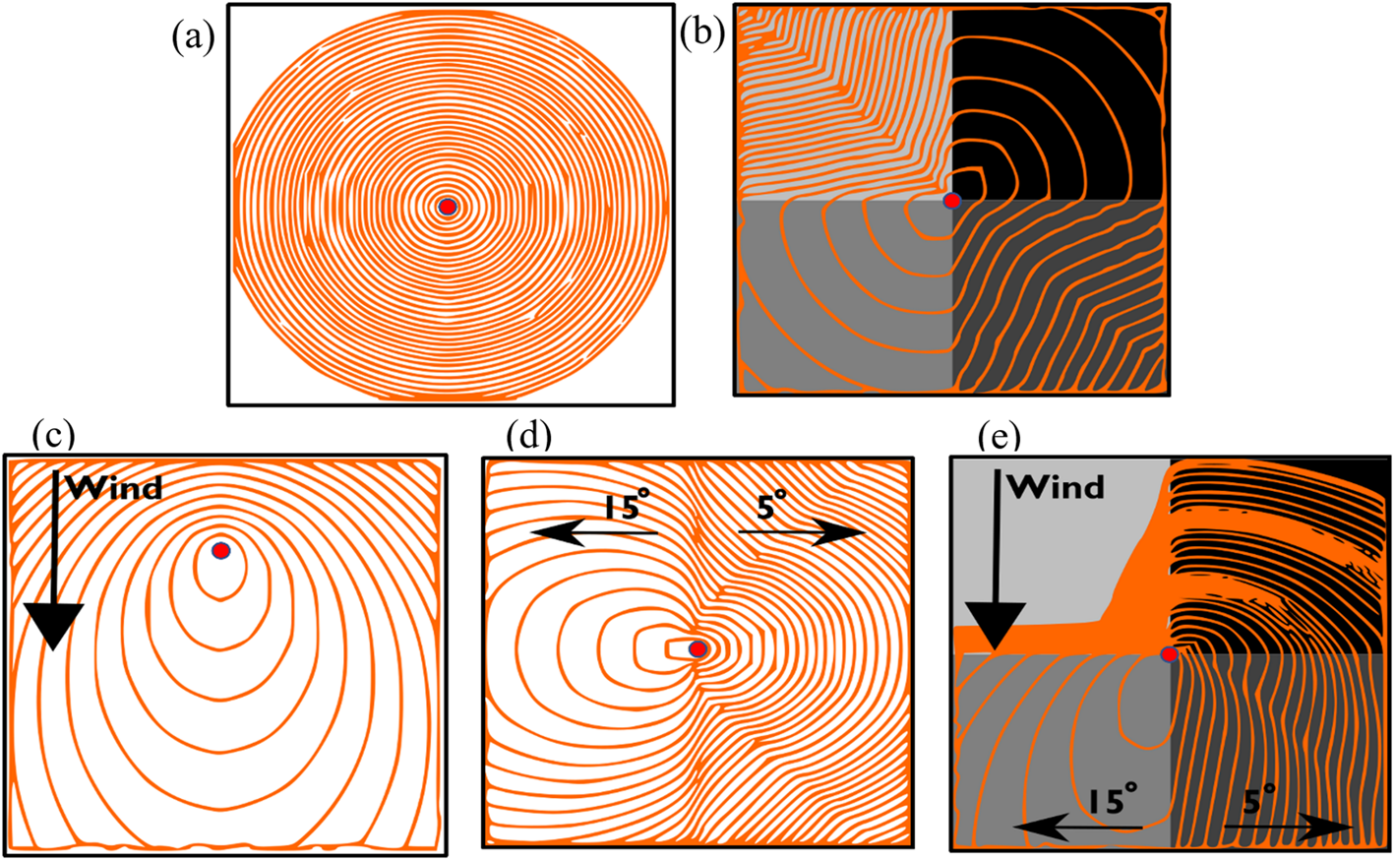
Visual representation of the tests for wildfire modelling. This includes predicted isochrones for fire spread for each test scenario. The ignition point is designated at the midpoint of the map. These scenarios also form the basis for the trigger buffer verification cases

This test refers to the evaluation of a trigger buffer model in case of homogenous characteristics. This means that this case (Fig. 2a) has flat topology (constant elevation and zero incline), uniform vegetation (fuel type, fuel moisture, canopy density, canopy height), spatially and temporally constant ambient temperature and humidity, and zero wind. For the TB1–TB5 verification tests, the populated regions are located at the midpoint of each quadrant of the map, each with an identical set evacuation time.

### Test B.2: varying fuel

This test refers to the evaluation of a trigger buffer model according to varying fuel. This means that the vegetation is different than the homogenous case (Fig. 2b) while the rest is the same as case B.1 (flat topology, spatially and temporally constant ambient temperature and humidity, and zero wind). The vegetation is divided into four identical quadrants, each with a different predefined fuel model, driving a different rate of spread in the wildfire model.

### Test B.3: set wind

The set wind case (Fig. 2c) includes a flat topology (constant elevation and zero incline), uniform vegetation (fuel type, fuel moisture, canopy density, canopy height), spatially and temporally constant ambient temperature and humidity. The wind is fixed at a constant speed, directed towards the south of the map, promoting spread of the wildfire model in the south, and impeding it towards the north end of the case map.

### Test B.4: valley

The valley case (Fig. 2d) includes uniform vegetation (fuel type, fuel moisture, canopy density, canopy height), spatially and temporally constant ambient temperature and humidity, and zero wind. The topography roughly forms a valley running through the centre of the map from north to south. Towards the east side of the map the land has a 5 degree elevation, and towards the west side of the map the land has a 15 degree elevation.

### Test B.5: heterogenous

The heterogenous case (Fig. 2e) includes vegetation divided into four identical quadrants, each with a different predefined fuel model, driving a different rate of spread in the wildfire model. The wind is fixed at a constant speed, directed towards the south of the map, promoting spread of the wildfire model in the south, and impeding it towards the north end of the case map. The topography roughly forms a valley running through the centre of the map from north to south. Towards the east side of the map the land has a 5 degree elevation, and towards the west side of the map the land has a 15 degree elevation. This case represents a complex environment that may be expected when modelling wildfire in a “real life” scenario.

A visual representation of the wildfire spread tests is presented in Fig. 2. Those tests are the basis for the trigger buffer tests (Mitchell 2019).

## Application of verification testing to WUI-NITY

An example application of the verification testing procedure has been performed with the WUI-NITY platform (Ronchi et al. 2020; Wahlqvist et al. 2021a) and its associated trigger buffer models (either PERIL (Mitchell 2019) or k-PERIL (Kalogeropoulos 2021)). The WUI-NITY platform simulates and visualises human behaviour and wildfire development in case of evacuation. It has been developed using a game engine (Unity 3D) so that wildfire spread, pedestrian response and movement, and traffic movement can be represented (Wahlqvist et al. 2021b). The WU-NITY platform is modular in that it allows for the coupling of different sub-models to estimate evacuation performance. The tool currently makes use of macroscopic approaches for the simulation of each modelling layer (i.e., fire, pedestrian, and traffic) and it is coupled with two trigger buffer models, PERIL, a-sub model run externally to WUI-NITY based on inputs from both the wildfire and evacuation sub models (Mitchell 2019), and k-PERIL, an updated trigger buffer model integrated into WUI-NITY (Kalogeropoulos 2021). In other words, while a wildfire is modelled, pedestrian response is represented along with their movement towards vehicles which are then accessing the traffic network. Routing is modelled through the implementation of the tool called Itinero (www.itinero.tech/) The readers are referred to the documents presenting WUI-NITY (Ronchi et al. 2021a; Wahlqvist et al. 2021b) and its associated trigger buffer model k-PERIL (Kalogeropoulos 2021; Mitchell et al. 2023) for a more detailed presentation of the modelling assumptions in use.

The verification tests were built in WUI-NITY by designing hypothetical simple scenarios in OpenStreetMap. JOSM^1^ was used to build these scenarios. JOSM is a free software editing tool for OpenStreetMap geodata created in Java. Table 3 presents the list of verification tests performed with WUI-NITY. They are currently grouped in relation to the type of layer they cover (pedestrian, traffic or integration with wildfire, including trigger buffers).

**Table 3:** List of verification tests performed, covering layer tested, core component, test code, test title, and sub-tests

Test code (layers) and core components	Test and sub-tests
*P.1 (pedestrian)* Population	Pedestrian re-distribution
*P.2 (pedestrian)* Population	Max number of available vehicles per household
*P.3 (pedestrian)* Pre-evacuation	*Response curve* Default Linear Custom
*P.4 (pedestrian)* Movement	*Pedestrian walking speed* Two values of walking speed (based on multipliers)
*PT.1 (pedestrian and traffic)* Movement	*Pedestrian distance to vehicles* Two values of walking speed (based on multipliers)
*T.1 (traffic)* Movement	*Uni-directional single-vehicle flow* T.1a: one road type T.1b: Multiple road types
*T.2 (traffic)* Movement Flow constraints	Background traffic
*T.3 (traffic)* Movement	*Change in carriageway configuration* Five traffic density levels (from no traffic to the jam-density, intended as flow equal to 0)
*T.4 (traffic)* Movement Flow constraints	*Relationships between speed–density and flow–density* Five traffic density levels (from no traffic to jam-density)
*T.5 (traffic)* Movement Flow constraints	*Vehicle speed reduction in reduced visibility conditions* Five traffic density levels (from no traffic to jam-density) Five optical density levels (0.05 m^−1^, 0.10 m^−1^, 0.15 m^−1^and 0.20 m^−1^)
*T.6 (traffic)* Movement Flow constraints	*Flow at destination* Five traffic density levels (from no traffic to jam-density)
*T.7 (traffic)* Movement Route/destination selection	*Group evacuation* No initial traffic Initial traffic density halfway the critical density
*T.8 (traffic)* Movement Flow constraints	Lane changing / overtaking
*T.9 (traffic)* Movement Flow constraints	Acceleration / deceleration
*T.10 (traffic)* Movement Flow constraints Event	Road crash
*T.11 (traffic)* Movement Flow constraints	Intersection
*T.12 (traffic)* Route/destination selection	Forced Destination
*T.13 (traffic)* Route/destination selection	*Destination choice in traffic* Fastest Closest Other conditions…
*T.14 (traffic)* Route/destination selection	*Route choice in traffic* Fastest Closest Other conditions…
*T.15 (traffic)* Movement Flow constraints	*Vehicle demand versus arrival distribution* Different numbers of vehicles
*WT.1 (wildfire and traffic)* Route/destination selection	Route loss
*WT.2 (wildfire and traffic)* Movement Event	Lane reversal
*WT.3 (wildfire and traffic)* Movement Flow constraints Event	Loss of exit or shelter
*WT.4 (wildfire and traffic)* Movement Flow constraints Event	Refuge capacity
*B.1 (wildfire and trigger buffer)* Base case (wildfire model) Base case for trigger buffers (PERIL) Base case for trigger buffers (k-PERIL)	Homogenous
*B.2 (wildfire and trigger buffer)* Varying fuel types (wildfire model) Varying fuel types for trigger buffers (PERIL) Varying fuel types for trigger buffers (k-PERIL)	Fuel types
*B.3 (wildfire and trigger buffer)* Varying wind (wildfire model) Varying wind for trigger buffers (PERIL) Varying wind for trigger buffers (k-PERIL)	Wind
*B.4 (wildfire and trigger buffer)* Topography (wildfire model) Topography for trigger buffers (PERIL) Topography for trigger buffers (k-PERIL)	Valley
*B.5 (wildfire and trigger buffer)* Fuel types, wind, topography (wildfire model) Fuel types, wind, topography (PERIL) Fuel types, wind, topography (k-PERIL)	Heterogenous

The detailed description of each test conducted is formatted in accordance with ISO 20414 (International Standards Organization 2020) and is reported in the supplementary material of this manuscript and in the openly accessible full report associated with this work (Ronchi et al. 2021a).

### Example of verification test for WUI-NITY

The application of these tests to the WUI-NITY model is presented to provide an example of a complete test and its associated results. This has been conducted according to the instructions provided in the test and results are presented according to the suggested reporting template. The example case presented here is test T.14 about route choice in traffic. Test T.14 was chosen as it also contains sub-tests so that an example of a full process could be shown.

#### Description of test T.14

*Name of the test* T.14 Route choice in traffic.

*Objective* Assess consistency between the conceptual implementation of route choice and model representation of route choice.

**Fig. 3:**
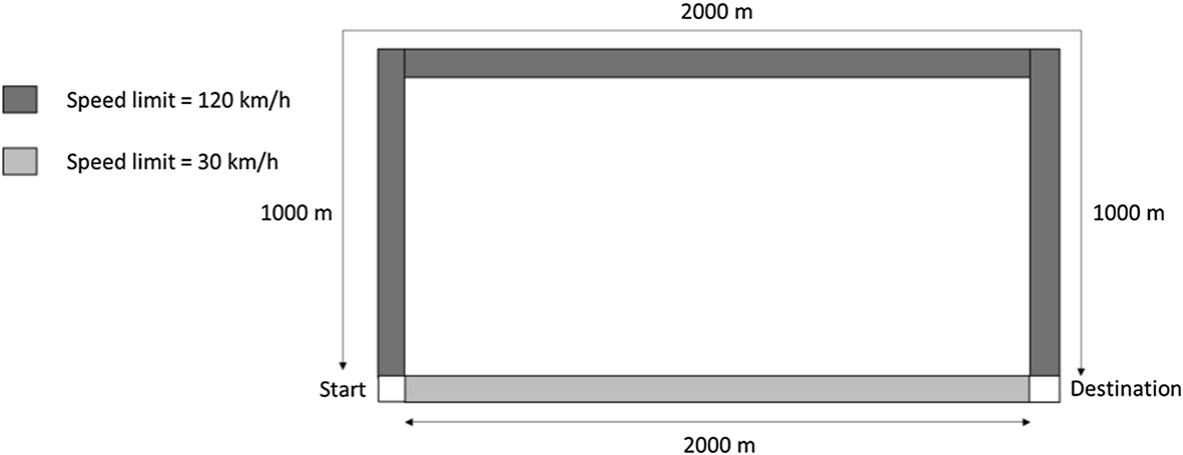
Geometric layout of scenario T.14

*Geometry* A road with a single carriageway considering movement on a single lane with a starting point connecting two separate roads leading to the same destination for a total length of either 4000 m or 2000 m (see Fig. 3). The road type for the longer route should correspond to a speed limit equal to 120 km/h. The road type for the shorter route should correspond to a speed limit equal to 30 km/h.

*Scenario(s)* One vehicle with an assigned free-flow speed corresponding to the speed limits (30 km/h and 120 km/h) moving along the road (from start to destination). Repeat the test for each destination choice method that is available (e.g., destination based on shortest route, fastest route, any other condition such as smoke that affects the selection). While running the test case, the user should turn off any non-relevant models, except the traffic simulation model.

*Expected result* The vehicle should drive to the appropriate route that corresponds to the route choice made and cover the distance of the road in the expected time (to be calculated in accordance with the modelling assumptions adopted).

*Test method* The test method is a quantitative verification of model results, i.e., the difference between the expected and the simulation results.

*User’s actions* The effectiveness of this test can be improved by setting additional prescriptions in relation to the type of model under consideration. The method for setting up the route should be reported.

#### Results of test T.14

The results obtained with WUI-NITY for test T.14 are reported according to the template presented in the verification testing protocol.

1. Does the model include a sub-model capable of representing the feature/behaviour included in the test?
  [x] Yes, feature/behaviour explicitly represented
  Route choice is computed by WUI-NITY, based on the open source *Itinero* tool.^2^ This was modified to allow route choice to be based on different criteria (e.g. fastest, shortest, affected by visibility conditions). The destinations of the vehicles within the traffic model are user-defined. Destination preferences can be set by the user, or by default, if there are several available destinations, the vehicles select the closest destination as an initial target. The user can also draw areas on the GUI for choosing destinations which override the default nearest option.
2. Geometry
  The road network is represented using JOSM in WUI-NITY, consisting of a road with a single carriageway and a single lane from a starting point connecting two separate roads leading to the same destination for a total length of either 4000 m or 2000 m. The road type for the longer route corresponded to a speed limit equal to 120 km/h. The road type for the shorter route corresponded to a speed limit equal to 30 km/h.
3. Scenario configuration
  One vehicle with an assigned free-flow speed corresponding to the road types drives toward the destination on the longer or shorter route depending on implemented route condition in WUI-NITY and is pre-defined by the user.
4. How have the behaviours been represented?
  [x] Explicitly: the model has a dedicated option to configure the relevant characteristics and response for this scenario.
5. Has the model tester performed a blind or open calculation?
  [x] Open
6. Did you run multiple simulations of the same scenario to produce the results?
  [x] NO
7. Did you repeat the test to study different configurations of this test?
  [x] YES
  The destination choice is changed between the available options of closest route or fastest route to the destination, smoke on the route or any other condition.
  The traffic model layer is the only one active during the testing.
  The simulation time step is 1 s.
8. Results
  The simulated result for the longer and faster route is 123 s.
  The expected result for the longer and faster route is 122 s (this calculated value considers the modelling assumptions adopted in the programme).
  The difference between the results is (123–122)/122 = 0.8%
  The simulated result for the shorter and slower route is 245 s.
  The expected result for the shorter and slower route in 243 s (this calculated value considers the modelling assumptions adopted in the programme).
  The difference between the results is (245–243)/243 = 0.8%

The simulation results match the expected results, with some small differences which are due to the time-step and the approximation adopted in the programming of the speed–density relationship.

### Results from verification tests

**Fig. 4:**
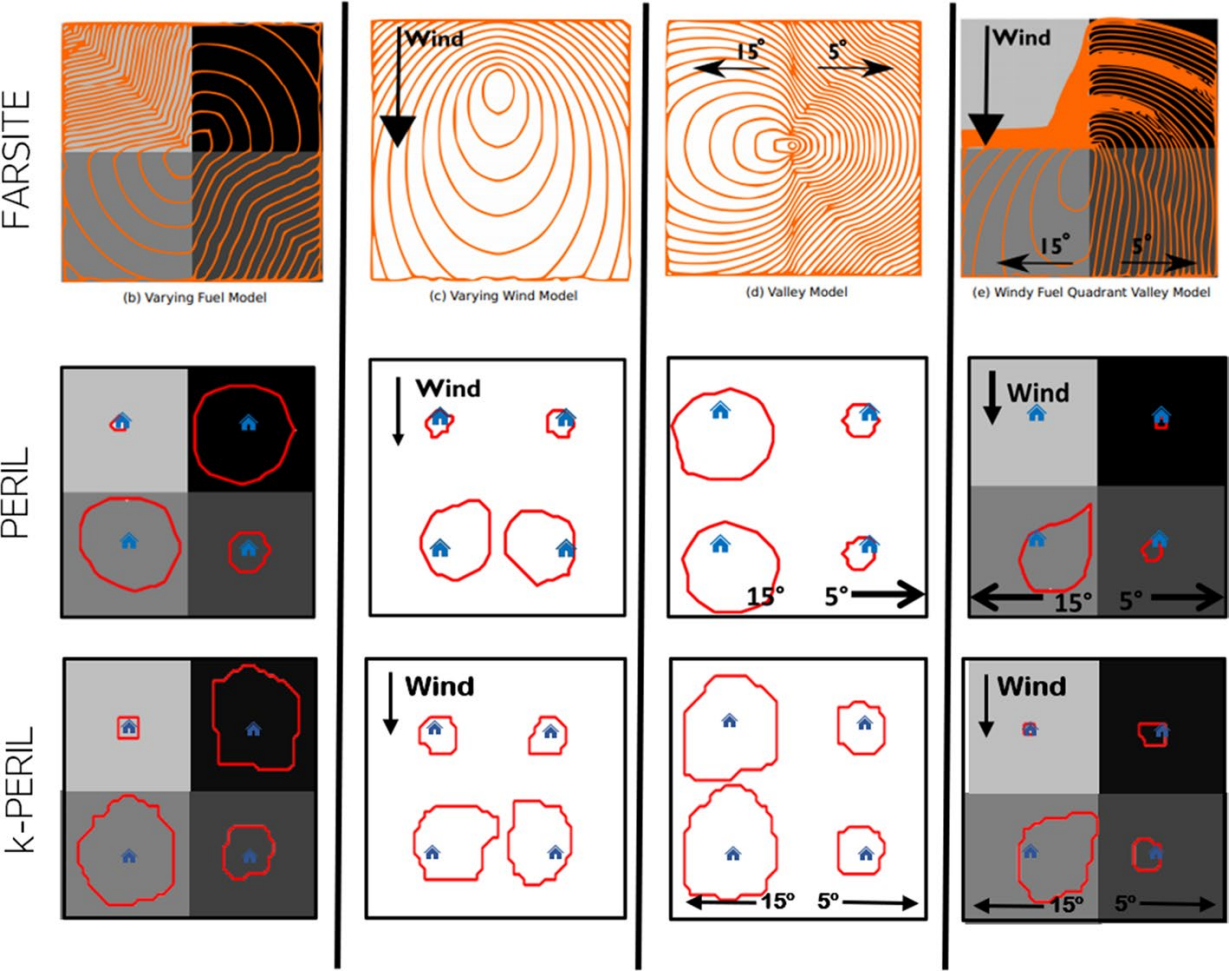
Visual representation of trigger buffer model tests B2–B5 (both PERIL and k-PERIL), based on the wildfire verification cases. Four test communities are positioned in each quadrant

The verification testing protocol was performed with WUI-NITY, following the same methodology as exemplified in the previous section. A short summary of the key findings that were obtained using WUI-NITY and PERIL/k-PERIL for the verification testing protocol is presented in Table 4. In addition, Fig. 4 presents a visual representation of the trigger buffer tests. The discussion includes which features worked in the model, which features could not be represented in the model, discrepancies in the results and speculations on the reasons that could have caused the discrepancies.

**Table 4:** Summary of results from the verification tests

Code	Title	Modelled explicitly?	
P.1	Pedestrian re-distribution	Y	
P.2	Max number of available vehicles per household	Y	
P.3	Response curve	Y	
P.4	Pedestrian walking speed	N	
	WUI-NITY does not include a model for evacuation movement on foot		
PT.1	Pedestrian distance to vehicle	Y	
	Small differences (< 2%) were observed due to the random placement of the population		
T.1a	Uni-directional single-vehicle flow (one road type)	Y	
	The difference for the 90 km/h configuration was 2%. This was caused by the approximation of the speed–density relationship equation implemented in the simulator		
T.1b	Uni-directional single-vehicle flow (multiple road types)	Y	
	There was a difference in results between simulation and hand-calculation of 0.9%. This was caused by the approximation of the speed–density relationship equation implemented in the simulator		
T.2	Background traffic	Y	
	There was a difference in results between simulation and hand-calculation of 1%. This was caused by the approximation of the speed–density relationship equation implemented in the simulator		
T.3	Change in carriageway configuration	Y	
	One of the configurations had a difference in results between simulation and hand-calculation. The difference for the density level 5 configuration was 6% due to the slightly different speed–density relationship in the simulator and hand calculations. In particular, the stall speed was approximated to 1.08 km/h in WUI-NITY rather than 1 km/h adopted in the hand calculations. The long runtime at this density level made this small difference in assumed speed more visible		
T.4	Speed–density and flow–density relationships	Y	
T.5	Vehicle speed reduction in reduced visibility conditions	Y	
	The observed differences were all below 3.3% due to a slight difference in stall speed. The long runtime at this density level made this small difference in assumed speed more visible		
T.6	Flow at destination	Y	
T.7	Group evacuation	N	
	WUI-NITY does not include a model for evacuation movement on foot		
T.8	Lane changing/overtaking	N	
	WUI-NITY adopts a macroscopic modelling approach		
T.9	Acceleration/deceleration	N	
	WUI-NITY adopts a macroscopic modelling approach		
T.10	Road crash	Y	
	One of the configurations had a difference in results between simulation and hand-calculation. The difference for the configuration with stall speed of 1 km/h in results between simulation and hand-calculation of 0.007. This was caused by the approximation of the speed–density relationship equation implemented in the simulator		
T.11	Intersection	Y	
	WUI-NITY adopts a macroscopic traffic modelling approach so only vehicles at aggregated levels are considered at intersections		
T.12	Forced Destination	Y	
T.13	Destination choice in traffic	Y	
	Slight differences were observed as the speed–density relationship was implemented in the simulator with some approximation		
T.14	Route choice in traffic (example case presented earlier)	Y	
	Both configurations had a difference in results between simulation and hand-calculation. The difference for the long route configuration was 0.8%. The difference for the short route configuration was 0.8%. This was caused by the approximation of the speed–density relationship equation implemented in the simulator		
T.15	Vehicle demand versus arrival distribution	Y	
WT.1	Route loss	N	
	A given route could be considered lost implicitly by blocking an adjacent destination		
WT.2	Lane reversal	Y	
	The difference in result for the fourth configuration was 1% due to the slightly different speed–density relationship equation implemented in the simulator and hand calculations		
WT.3	Loss of exit or shelter	Y	
WT.4	Refuge capacity	Y	
B.1	Wildfire and Trigger Buffer – Homogenous	Y	
	Uniform wildfire isochrones given by wildfire model and approximately uniform trigger buffer perimeters from PERIL and k-PERIL. Slight difference between PERIL and k-PERIL for TB1-TB5 due to faster but less accurate shortest path algorithm used		
B.2	Wildfire and Trigger Buffer – Fuel Types	Y	
	Wildfire isochrones and trigger buffers of different sizes in each quadrant depending on fuel type		
B.3	Wildfire and Trigger Buffer – Wind	Y	
	Elliptical wildfire isochrones larger in the direction of wind. Trigger buffers larger in the south compared to the north		
B.4	Wildfire and Trigger Buffer—Valley	Y	
	Complex wildfire isochrones and trigger buffers, both larger in west compared to east		
B.5	Wildfire and Trigger Buffer—Heterogeneous	Y	
	Complex wildfire isochrones and trigger buffers		

*Y* Yes, *N* No

## Discussion

This paper introduced a new verification testing protocol specifically designed for wildfire evacuation models. Verification tests, such as the ones provided here, are fundamental steps towards reliable model results. This is particularly relevant for wildfire evacuation models, as the complexity of the interactions between modelling layers need to be scrutinized to ensure accuracy in the results produced. This paper presents an overview of the verification tests, the readers are referred to the supplementary material and the full report associated with this paper for the details of the testing protocol (Ronchi et al. 2021a).

The verification testing protocol was applied to the openly available tool WUI-NITY and its associated trigger buffer model k-PERIL. Most of the proposed verification tests could be conducted within WUI-NITY except those that required a microscopic modelling approach. It should be noted that the current verification testing protocol focusses primarily on evacuation via private vehicles rather than on other transportation modes (e.g., public transport, or transport via air/sea). This choice was made since private vehicles are the most common transportation mode in wildfire evacuation scenarios. In addition, in this first attempt to produce a verification protocol, the focus was mainly on the basic features represented with macroscopic modelling approaches. This led to a set of simplifications, such as the assumption that free flow speed corresponds to the speed limit when testing a model. Future efforts should be placed in expanding the protocol with more sophisticated tests for microscopic modelling features (e.g., signalized intersections in the traffic component, more advanced speed choice modelling). In addition, the testing protocol is designed for testing different components in isolation along with a set of tests which address more than one modelling layer. Future research can focus on expanding the protocol to make sure that two (or more) tests pass verification while run together.

For the traffic component, the difference between simulation results compared with hand calculations ranged from 0 to 8%. Approximations in the implementation of the speed–density relationship caused relatively small differences in speeds used in the simulation, thus affecting test results. Those differences were not observed in the tests in which a complete agreement in the representation of speeds between hand calculations and simulations had been observed. The reason for these similarities could be linked to time-step approximations errors that made the difference negligible. In the tests with smaller differences in results, the overall test run time was shorter because of higher driving speeds, which resulted in a lower number of time-steps. In the tests with larger differences, the run time was generally longer due to vehicles encountering congested conditions. The difference in speeds obtained in the simulation versus the hand calculations was negligible (≈ 0.07 km/h), but in a test with several time steps, the impact of the speed difference became more prominent. This observation suggests that model accuracy can decrease as the number of calculated time-steps increases.

The wildfire model verification showed identical isochrones across B1-B5 between FARSITE (Finney et al. 1998) and WUI-NITY. FARSITE is a wildfire spread model for which verification cases are widely available online and its results can be imported into WUI-NITY.

Regarding the testing of PERIL and k-PERIL (see Table 4), the results related to the homogenous flat land (B.1) showed a uniform spread rate regardless of direction, thus resulted in the expected uniform circle around the community (see Fig. 4). Considering more flammable fuel types, as expected, the radius from the community increased, due to the fire approaching the community at a greater rate of spread. The impact of wind and topography also worked as intended. In the verification cases for trigger buffers, the slope is shown to have great impact on the trigger perimeters. Testing also showed that more complex trigger perimeters can be expected from inputs of features reflecting heterogenous fire scenarios. In general, thicker boundaries were formed along the directions of greater fire spread, as expected. The results of k-PERIL also closely match those of PERIL, with small differences contributing to the faster yet less accurate shortest path algorithm.

All in all, WUI-NITY and PERIL/k-PERIL performed as expected. It should be noted that it is here left up to the model user to judge if a given testing result is acceptable or not. This approach is in line with current practice for other verification protocols (e.g., in ISO 20414 (International Standards Organization 2020), where no acceptance criteria are specifically provided along with the testing protocol. This decision is taken since the choice of acceptance criteria will largely depend on the specific application wildfire evacuation cases and the information available for the model input calibration. The idea behind referring to ISO 20414 as a starting point for developing the current testing protocol was to adopt a methodological approach which is familiar to fire safety engineers/managers. While this could create issues with acceptance within the transportation modelling world (which may be familiar with other methods and approaches), this choice is deemed appropriate given the users of the tools under consideration.

There are number of limitations that need to be acknowledged in this work. First, the completeness of verification tests is unclear. It is possible that future model developers will require a wider range of verification tests than the ones presented here. Since this the first attempt at developing a verification protocol for wildfire evacuation models, a deliberate choice was made to keep the number of tests to a manageable size, so that future model developers, users and testers are encouraged to run the protocol in full. For example, policy responses have not been considered comprehensively (e.g., only lane reversal/contraflow has been considered). In addition, the current verification protocol has been developed following the approach of ISO 20414 (International Standards Organization 2020), which has served as reference document concerning the number of tests to be provided and their nature. Second, the verification protocol is primarily designed for macroscopic tools. Future expansion of these tests should allow for a finer grained, mesoscopic or microscopic modelling representation of all modelling layers. In this context, a transition to microscopic simulations for all layers would require potentially more and different verification tests. Fourth, while we present an extensive list of tests, they are mostly focused on traffic modelling and to a lesser extent on trigger buffer, pedestrian, and fire modelling. Future work is needed to expand this list to consider other modelling layers in full and particularly their interactions between each other.

Finally, verification is only half of the V&V process. Equally important is the aspect of model *validation*. While beyond the scope of the present paper, future research efforts are clearly needed to provide empirical data for validation purposes. Data collection efforts should ideally consider all modelling layers and core components included in wildfire evacuation modelling tools. Unfortunately, the lack of data concerning evacuation behaviour is a known issue in the field of wildfire hazards (Kuligowski 2020). Existing data refer to evacuation at different levels such as decision-making such as the response-phase/evacuation decision, e.g., (McCaffrey et al. 2018; Lovreglio et al. 2019; Kuligowski et al. 2020, 2022; Grajdura et al. 2021; Zhao et al. 2022; Katzilieris et al. 2022b), traffic dynamics e.g., (Hou et al. 2022; Rohaert et al. 2023), and route/destination choice, e.g., (Toledo et al. 2018; Wong 2020; Wong et al. 2020, 2022). Once there are sufficient data to include validation in the testing of wildfire evacuation models, this could be developed following the same approach as the proposed verification protocol. To be sufficient, there should be a body of validation datasets that can cover all the key features/sub-models of wildfire evacuation models, include a variety of data (both stated preferences and revealed preferences) and be collected with different approaches to compensate strengths and weaknesses of different data collection methods. Existing datasets and analyses are generally focused on individual events (with rare exceptions such as (Hou et al. 2022), which present specific characteristics related to the population and the scenario conditions in which they took place. For instance, they mostly address evacuation via private vehicles (e.g., limited literature is available on the use of other evacuation modes (Filippidis et al. 2020)) and they mostly refer to regions which have experienced wildfire-related issues for several years. For a validation protocol to be comprehensive and allow a global usage of wildfire evacuation models, it would have to include a variety of datasets which cover the whole range of populations, transportation modes and scenario conditions which may occur. The risk with providing a validation protocol which is potentially incomplete is to be able to provide only partial validation (while calling it model validation intending the whole system), by just testing it with a handful of data on very specific features, transportation modes, populations and wildfire evacuation scenarios. The current effort on verification testing is a needed starting point to ensure that the basic features/sub-models of wildfire evacuation models work as intended.

## Conclusion

The present work proposes a set of practical verification test scenarios for wildfire evacuation models and illustrates their application. The integration of these or similar tests into wildfire evacuation modelling is deemed to be an essential step to test and establish the credibility of modelling tools used to investigate wildfire safety. If integrated in the development process of wildfire evacuation tools, practitioners can be provided with transparent and credible evidence with regard to the verification of the models they use.

## Supplementary Information

Below is the link to the electronic supplementary material.

Supplementary file1 (DOCX 250 kb)

## Footnotes

1 https://josm.openstreetmap.de/.

2 https://www.itinero.tech/.
