# Supplementary material for: The verification of wildland–urban interface fire evacuation models
Source: Nat Hazards (Dordr). 2023 Mar 28;117(2):1493–519. doi: 10.1007/s11069-023-05913-2 (PMC10220130; doi:10.1007/s11069-023-05913-2)
Supplement: Supplementary file 1 — Supplementary file1 (DOCX 250 kb) [file 11069_2023_5913_MOESM1_ESM.docx]

**Appendix A – List of verification tests**

| **P.1** | **Pedestrian re-distribution** |
| --- | --- |
| Objective | Assess consistency between the conceptual and implemented re-distribution of pedestrians in space based on available routes |
| Geometry | A walkable area of 1000 m * 1000 m including a set of households. 50% of those households have access to a node of the road network, the rest does not have access (Figure A.1 shows an example).  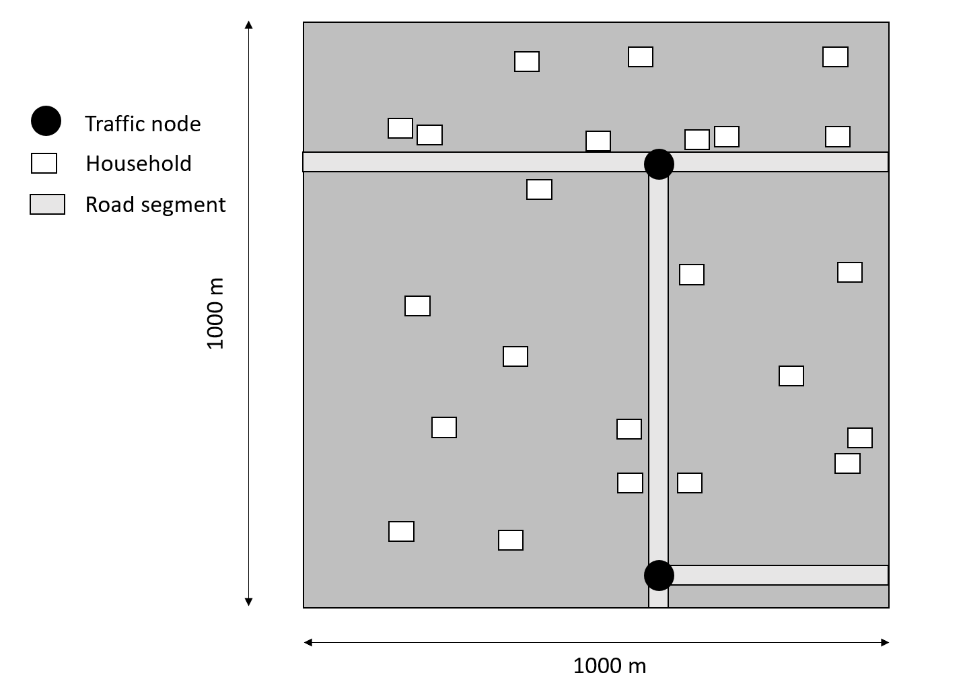  *Figure A.1. Geometry of scenario P.1.* |
| Scenario(s) | Let the simulator re-distribute people in the households based on the algorithm adopted by the model, so that all population can access a node of the road network. |
| Expected result | All of the population should be able to access the road network and the number of people accessing the road network should correspond to the number of people implemented in the scenario. |
| Test method | The test method is a quantitative verification of model results, i.e. the difference between the expected result and the simulation results. |
| User’s actions | The effectiveness of this test can be improved by setting additional prescriptions in relation to the type of model under consideration. For example, in the case of models that use a network approach, results may be dependent on the configuration of the network/grid adopted. For grid-based models, considerations should also be made by the tester on the necessity of performing this test with different configurations (e.g. simulating the default cell size and a set of both reduced and increased cell sizes) in order to test the sensitivity of the results to cell size. The method for setting up the population re-distribution should be reported. |

| **P.2** | **Max number of available vehicles per household** |
| --- | --- |
| Objective | Assess consistency between implemented relationship for number of vehicles distributed to each household and hand-calculated results. |
| Geometry | A walkable area considering movement to a traffic node (example in Figure A.2).  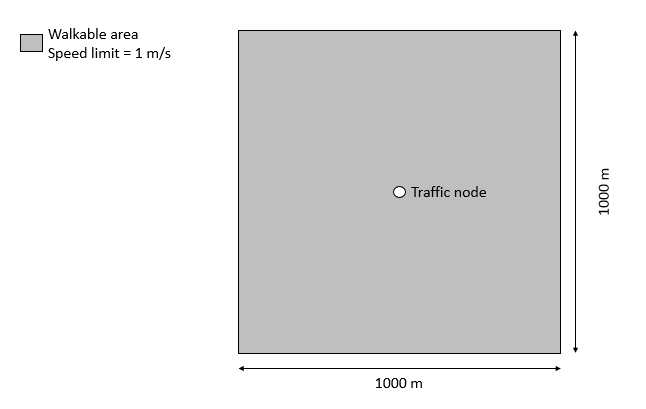  *Figure A.2. Geometry of scenario P.2.* |
| Scenario(s) | A given group of pedestrians leave their households (moving towards vehicles in a traffic node) with an assigned movement speed of 1 m/s and a response time equal to 0 moving along the walkable area to the traffic node. Each household should have 1-5 vehicles available given predetermined values. While running the test case, the user should turn off any non-relevant models, except the pedestrian simulation model. |
| Expected result | The number of vehicles entering the traffic model should correspond to the implemented numbers of vehicles assigned to each household with its probability distribution for additional vehicles (to be calculated in accordance with the modelling assumptions adopted). |
| Test method | The test method is a quantitative verification of model results, i.e. the difference between the expected result and the simulation results. |
| User’s actions | The effectiveness of this test can be improved by setting additional prescriptions in relation to the type of model under consideration. For example, in the case of models that use a network approach, results may be dependent on the configuration of the network/grid adopted. For grid-based models, considerations should also be made by the tester on the necessity of performing this test with different configurations (e.g. simulating the default cell size and a set of both reduced and increased cell sizes) in order to test the sensitivity of the results to cell size. |

| **P.3** | **Response curve** |
| --- | --- |
| Objective | Assess consistency between implemented pedestrian response model and hand-calculated results. This includes comparing the number of pedestrians that evacuate before the evacuation alarm, after the alarm and those who do not evacuate. |
| Geometry | A walkable area of 1000 m * 1000 m, which includes a set of (sufficiently large) households defined by the user (example in Figure A.3).  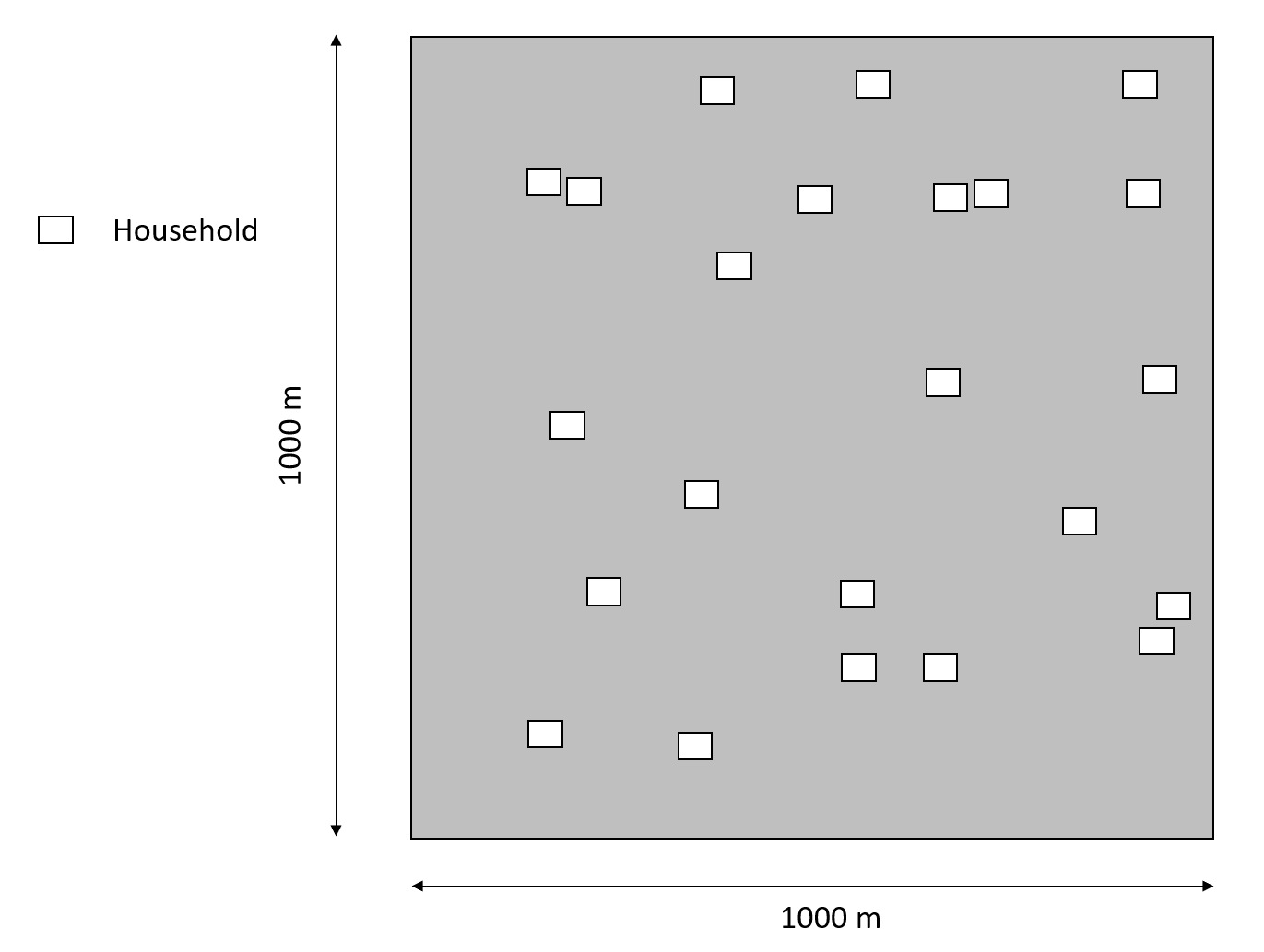  *Figure A.3. Geometry of scenario P.3.* |
| Scenario(s) | A given group of pedestrians leave their households with a distributed response time drawn from the default response curve. Repeat the test using a custom response curve for the pedestrians on the walkable area (e.g. using a linear or custom response curve, starting on X-axis < 0). While running the test case, the user should turn off any non-relevant models, except the pedestrian response model. |
| Expected result | The pedestrians should leave their households in accordance with the expected time and the % of people evacuating before the trigger, after the trigger and those who do not evacuate should correspond to the pre-defined response curve (to be calculated in accordance with the modelling assumptions adopted). |
| Test method | The test method is a quantitative verification of model results, i.e. the difference between the expected result and the simulation results. |
| User’s actions | The test should be repeated for each distribution type included in the model. |

| **P.4** | **Pedestrian walking speed** |
| --- | --- |
| Objective | Assess consistency between the conceptual and implemented relationship concerning pedestrian movement based on walking speeds. |
| Geometry | A walkable area of 1000 m * 1000 m considering movement for a total length of 1000 m and assuming no obstacles along the path (example in Figure A.4). The walkable area should correspond to a speed limit equal to 1 m/s.  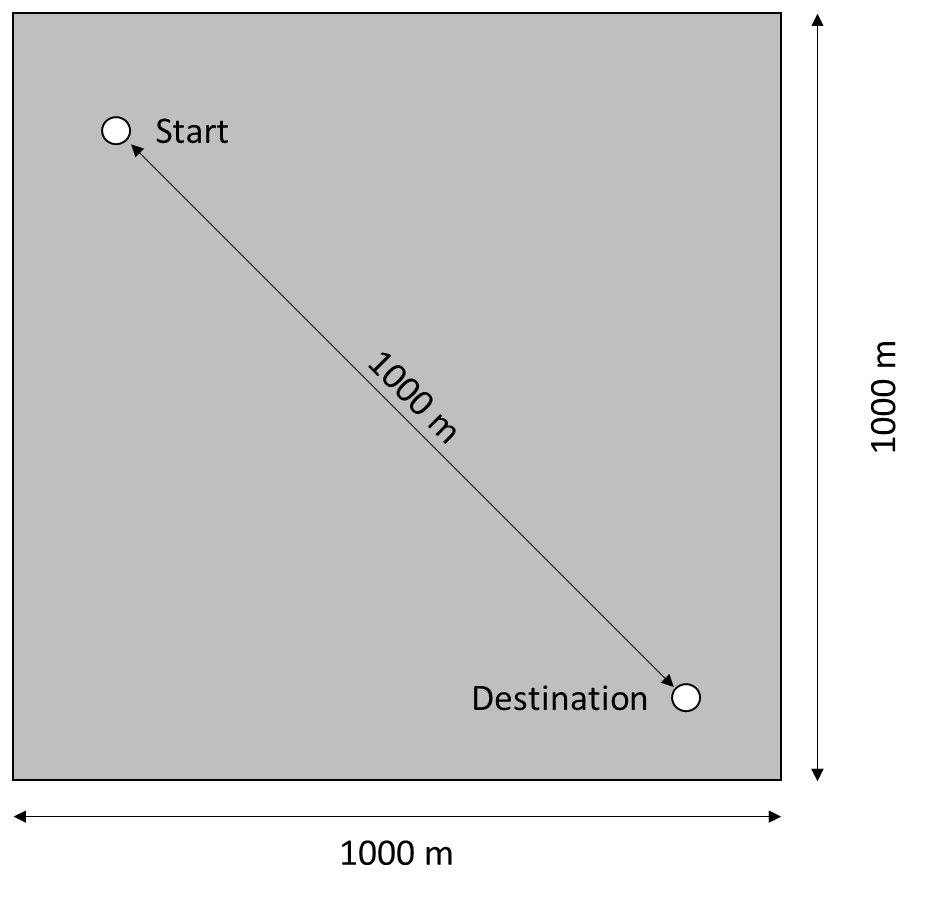  *Figure A.4. Geometry of scenario P.4.* |
| Scenario(s) | One pedestrian with an assigned movement speed of 1 m/s and a response time equal to 0 s moving along the walkable area (from start to destination), with a given speed multiplier. Repeat the test varying the speed multiplier of pedestrian walking speed on the walkable area (e.g., using 2 different values of speed multipliers from no multiplier (=1) to a multiplier that corresponds to a pedestrian that adopts a slower walking speed (< 1)). While running the test case, the user should turn off any non-relevant models, except the pedestrian simulation model. |
| Expected result | The pedestrian should cover the distance to the traffic node in expected time (to be calculated in accordance with the modelling assumptions adopted). |
| Test method | The test method is a quantitative verification of model results, i.e. the difference between the expected result and the simulation results. |
| User’s actions | The effectiveness of this test can be improved by setting additional prescriptions in relation to the type of model under consideration. For example, in the case of models that use a network approach, results may be dependent on the configuration of the network/grid adopted. For grid-based models, considerations should also be made by the tester on the necessity of performing this test with different configurations (e.g. simulating the default cell size and a set of both reduced and increased cell sizes) in order to test the sensitivity of the results to cell size. The method for setting up the destination should be reported. The model tester should also report if the pedestrian exhibits acceleration/deceleration during the movement. |

| **PT.1** | **Pedestrian distance to vehicle** |
| --- | --- |
| Objective | Assess consistency between implemented relationship between pedestrian distance and hand-calculated results. |
| Geometry | A walkable area of 100 m * 100 m considering movement for a total length of 100 m (example in Figure A.5). The walkable area should correspond to a speed limit equal 1 m/s.  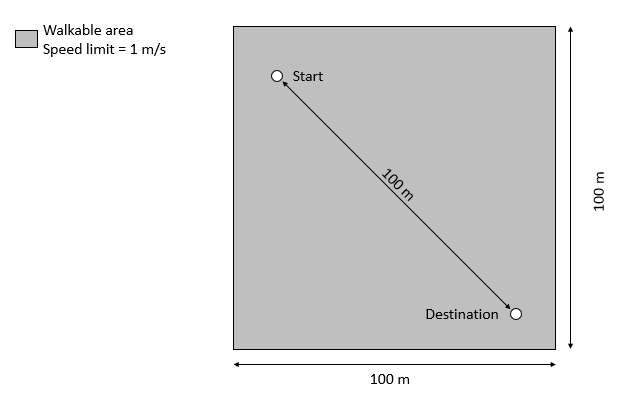  *Figure A.5. Geometry of scenario PT.1.* |
| Scenario(s) | One pedestrian with an assigned movement speed of 1 m/s and a response time equal to 0 s moving along the walkable area (from start to destination), with a given distance multiplier. Repeat the test varying the distance multiplier of pedestrian distance on the walkable area (e.g., using 2 different values of distance multiplier from no multiplier (=1) to a multiplier that corresponds to a pedestrian taking a route with movement inefficiency (>1)). While running the test case, the user should turn off any non-relevant models, except the pedestrian simulation model and the traffic network. |
| Expected result | The pedestrian should cover the distance to the traffic node in expected time (to be calculated in accordance with the modelling assumptions adopted). |
| Test method | The test method is a quantitative verification of model results, i.e. the difference between the expected result and the simulation results. |
| User’s actions | The effectiveness of this test can be improved by setting additional prescriptions in relation to the type of model under consideration. For example, in the case of models that use a network approach, results may be dependent on the configuration of the network/grid adopted. For grid-based models, considerations should also be made by the tester on the necessity of performing this test with different configurations (e.g. simulating the default cell size and a set of both reduced and increased cell sizes) in order to test the sensitivity of the results to cell size. The method for setting up the destination should be reported. The model tester should also report if the pedestrian exhibits acceleration/deceleration during the movement. |

| **T.1a** | **Uni-directional single vehicle flow (one road type)** |
| --- | --- |
| Objective | Assess consistency between speed assignment of one vehicle on a single road type and model representation for uni-directional movement. |
| Geometry | A road with a single carriageway considering movement on a single lane for a total length of 1000 m (example in Figure A.6). The road type should correspond to a speed limit equal to 90 km/h.  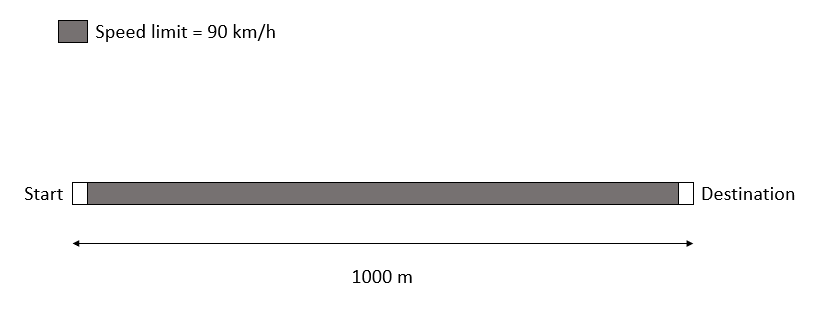  *Figure A.6. Geometry of scenario T.1a.* |
| Scenario(s) | One vehicle with an assigned free flow speed corresponding to the speed limit (90 km/h) moving along the road (from start to destination). Repeat the test changing the road type to correspond to a speed limit on the lower end (e.g. 30 km/h) as well as a speed limit on the higher end (e.g. 120 km/h). While running the test case, the user should turn off any non-relevant models, except the traffic simulation model. |
| Expected result | The vehicle should cover the distance of the road in the expected time (to be calculated in accordance with the modelling assumptions adopted) |
| Test method | The test method is a quantitative verification of model results, i.e. the difference between the expected result and the simulation results. |
| User’s actions | The effectiveness of this test can be improved by setting additional prescriptions in relation to the type of model under consideration. For example, in the case of models that use a network approach, results may be dependent on the configuration of the network/grid adopted. For grid-based models, considerations should also be made by the tester on the necessity of performing this test with different configurations (e.g. simulating the default cell size and a set of both reduced and increased cell sizes) in order to test the sensitivity of the results to cell size. The method for setting up the destination should be reported. The model tester should also report if the vehicle exhibits acceleration/deceleration during the movement. |

| **T.1b** | **Uni-directional single vehicle flow (multiple road types)** |
| --- | --- |
| Objective | Assess consistency between speed assignment of one vehicle on multiple road types and model representation for uni-directional movement. |
| Geometry | A road with a single carriageway considering movement on a single lane for a total length of 1000 m + 1000 m (see Figure A.7) with an unsignalised intersection (the user can choose the length of the intersecting road, in this example this is equal to 1000 m). The first part includes a road type corresponding to a speed limit equal to 50 km/h, the second part a speed limit equal to 90 km/h.  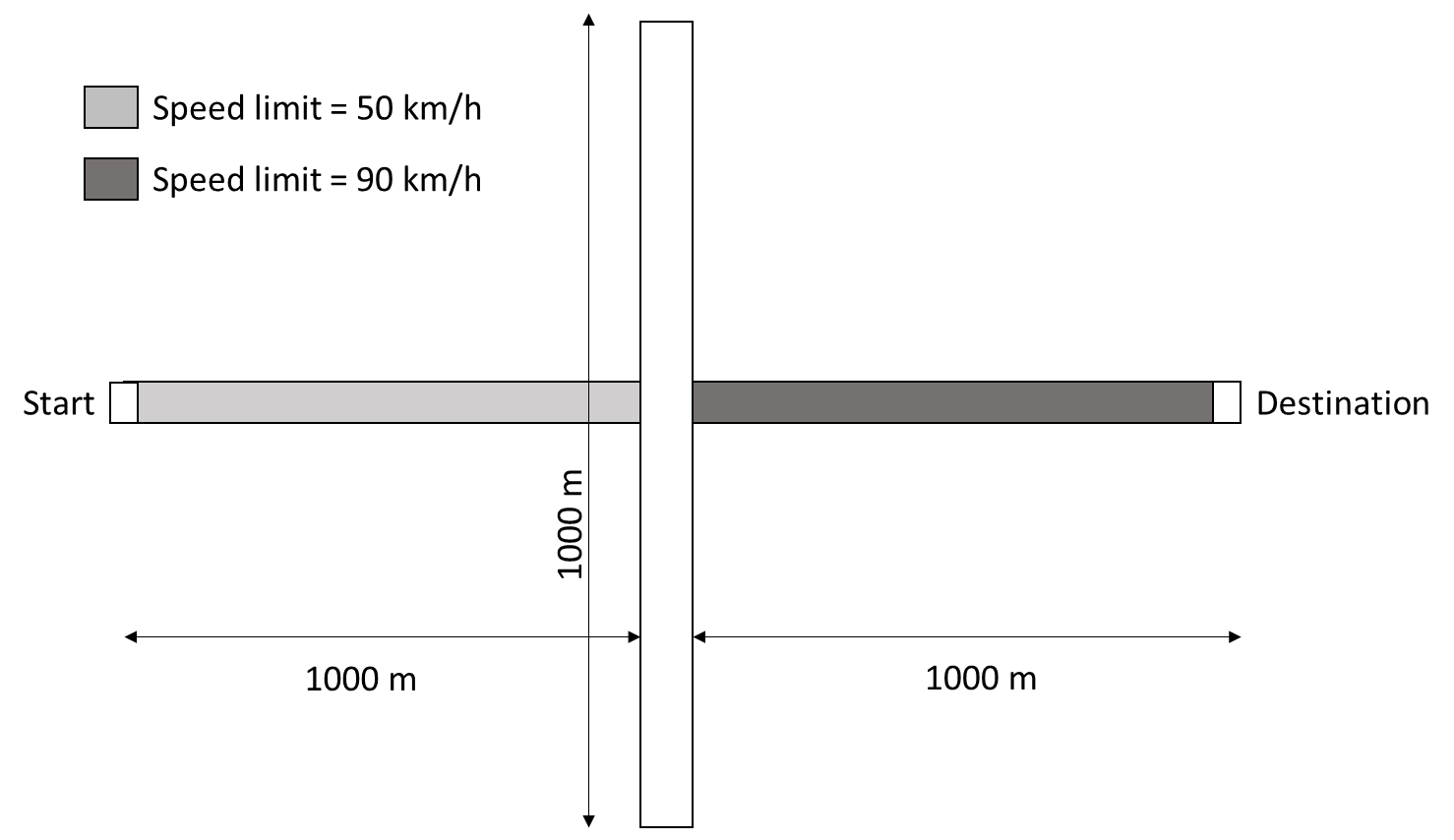  *Figure A.7. Geometry of scenario T.1b.* |
| Scenario(s) | One vehicle with an assigned free flow speed corresponding to the speed limits (50 km/h and 90 km/h) moving along the road (from start to destination). While running the test case, the user should turn off any non-relevant models, except the traffic simulation model. |
| Expected result | The vehicle should cover the distance of the road in the expected time (to be calculated in accordance with the modelling assumptions adopted) |
| Test method | The test method is a quantitative verification of model results, i.e. the difference between the expected result and the simulation results. |
| User’s actions | The effectiveness of this test can be improved by setting additional prescriptions in relation to the type of model under consideration. For example, in the case of models that use a network approach, results may be dependent on the configuration of the network/grid adopted. For grid-based models, considerations should also be made by the tester on the necessity of performing this test with different configurations (e.g. simulating the default cell size and a set of both reduced and increased cell sizes) in order to test the sensitivity of the results to cell size. The method for setting up the destination should be reported. The model tester should also report if the vehicle exhibits acceleration/deceleration during the movement. |

| **T.2** | **Background traffic** |
| --- | --- |
| Objective | Ensure the impact of background traffic on vehicle flow is correctly implemented. |
| Geometry | A road with a single carriageway considering movement on a single lane for a total length of 1000 m (see Figure A.8). The road type should correspond to a speed limit equal to 90 km/h.  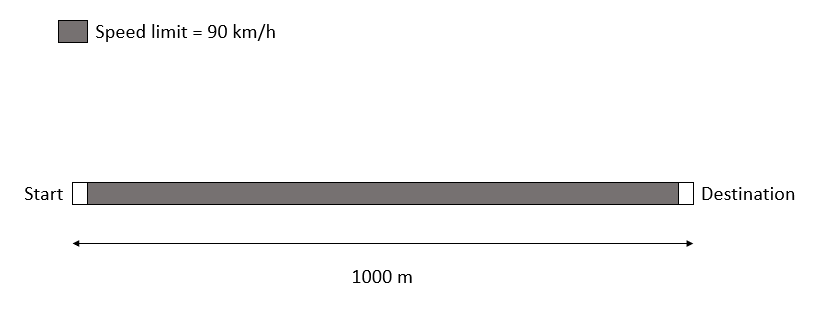  *Figure A.8. Geometry of scenario T.2.* |
| Scenario(s) | One vehicle with an assigned free flow speed corresponding to the speed limit (90 km/h) moving along the road (from start to destination). A background traffic reducing the flow capacity of the road of 50% is implemented. The 50% of reduction due to background traffic is an example to test the capability of the model of representing background traffic. Any value of reduced flow can be represented in the model (based on the current loading of the network). While running the test case, the user should turn off any non-relevant models, except the traffic simulation model. |
| Expected result | The vehicle should cover the distance of the road in the expected time (to be calculated in accordance with the modelling assumptions adopted) |
| Test method | The test method is a quantitative verification of model results, i.e. the difference between the expected result and the simulation results. |
| User’s actions | The effectiveness of this test can be improved by setting additional prescriptions in relation to the type of model under consideration. For example, in the case of models that use a network approach, results may be dependent on the configuration of the network/grid adopted. For grid-based models, considerations should also be made by the tester on the necessity of performing this test with different configurations (e.g. simulating the default cell size and a set of both reduced and increased cell sizes) in order to test the sensitivity of the results to cell size. The method for setting up the destination should be reported. The model tester should also report if the vehicle exhibits acceleration/deceleration during the movement. |

| **T.3** | **Change in carriageway configuration** |
| --- | --- |
| Objective | Assess consistency between the implemented impact of change in carriageway configuration and the calculated impact for uni-directional movement. |
| Geometry | A road with a single carriageway for a total length of 1000 m + 1000 m (see Figure A.9) with an unsignalised intersection (the user can choose the length of the intersecting road, in this example this is equal to 1000 m) is considered. The initial 1000 m segment of the road has one lane per carriageway, while the following 1000 m segment has two lanes per carriageway. The road type should correspond to a speed limit equal to 90 km/h.  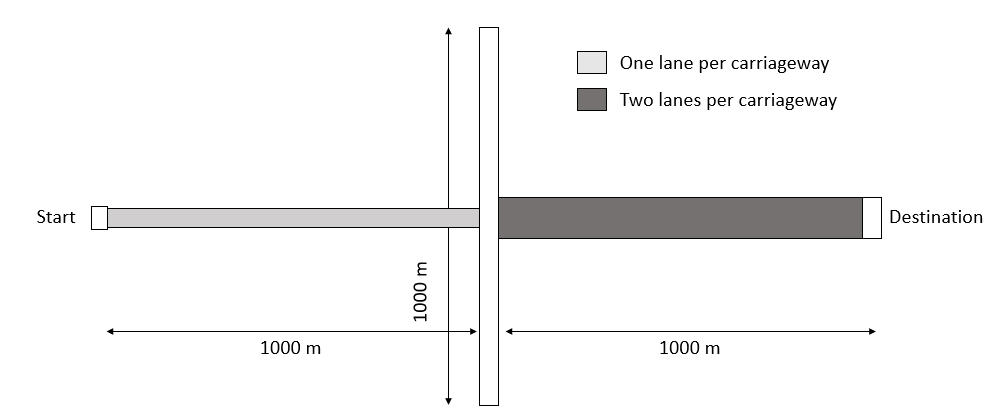  *Figure A.9. Geometry of scenario T.3.* |
| Scenario(s) | One vehicle with an assigned free flow speed corresponding to the speed limit (90 km/h) moving along the road (from start to destination). Repeat the test varying the initial density of vehicle on the road (e.g. using 5 vehicle density levels linearly from 1 veh/km/lane to the vehicle density corresponding to the vehicle being stopped considering the portion of the road with smaller capacity). The associated speed limit in the two road segments changes accordingly. While running the test case, the user should turn off any non-relevant models, except the traffic simulation model. |
| Expected result | The vehicle should cover the distance of the road in the expected time (to be calculated in accordance with the modelling assumptions adopted) |
| Test method | The test method is a quantitative verification of model results, i.e. the difference between the expected result and the simulation results. |
| User’s actions | The effectiveness of this test can be improved by setting additional prescriptions in relation to the type of model under consideration. For example, in the case of models that use a network approach, results may be dependent on the configuration of the network/grid adopted. For grid-based models, considerations should also be made by the tester on the necessity of performing this test with different configurations (e.g. simulating the default cell size and a set of both reduced and increased cell sizes) in order to test the sensitivity of the results to the cell size. The method for setting up the destination should be reported. The model tester should also report if the vehicle exhibits acceleration/deceleration during the movement. |

| **T.4** | **Relationships between speed-density and flow-density** |
| --- | --- |
| Objective | Assess qualitative consistency between the implemented relationships between traffic flow/density and speed/density in a road segment and simulated one considering uni-directional movement. |
| Geometry | A road segment is represented with a single carriageway considering movement on a single lane for a total length of 3000 m divided in three zones of equal length (see Figure A.10). The road type should correspond to a speed limit equal to 70 km/h. The road segment is divided in three zones, namely zone 1 (white), zone 2 (light grey) and zone 3 (white).  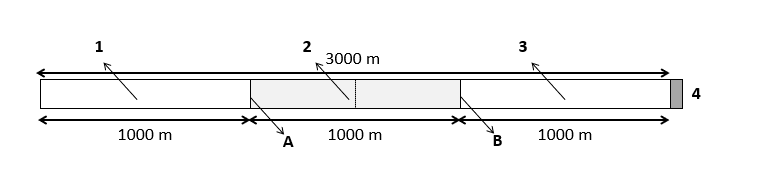  *Figure A.10. Geometry of scenario T.4. The drawing is off scale to facilitate visualization.* |
| Scenario (s) | Calculate 5 vehicle density levels linearly ranging from 1 veh/km/lane to the vehicle density corresponding to a congested scenario (Density 1=D1=1 veh/km/lane, D2, D3, D4 and D5=density leading to stopped vehicles on the road segment). The vehicles are uniformly distributed in the entire road segment (Zones 1, 2 and 3). They have an initial free flow speed equal to the speed limit.  Step 1: Assign a number of vehicles corresponding to the D3 vehicle density on the road segment to move to the right towards the destination of the road segment. Place the last vehicle in Zone 2 near line A and measure the time that it takes from line A to line B and estimate the associated driving speed. Measure the average vehicle flows in line B (with a time interval decided by the tester) starting from the beginning of the simulation until the last vehicle in zone 2 arrives to Line B. Vehicle densities in Zone 2 are recorded when the last vehicle in Zone 2 reaches the centre of Zone 2.  Step 2: Step one is repeated with D1, D2, D4 and D5.  While running the test case, the user should turn off any non-relevant models, except the traffic simulation model. |
| Expected result | The relationship between driving speeds and vehicle densities in Zone 2 as well as the flows across Line A vs vehicle densities in Zone 2 are plotted and compared with the underlying assumptions used in the traffic evacuation model. |
| Test method | The test method is a qualitative verification of the vehicle movement. |
| User’s actions | The effectiveness of this test can be improved by setting additional prescriptions in relation to the type of model under consideration. For example, in the case of models that use a network approach, results may be dependent on the configuration of the network/grid adopted. For grid-based models, considerations should also be made by the tester on the necessity of performing this test with different configurations (e.g. simulating the default cell size and a set of both reduced and increased cell sizes) in order to test the sensitivity of the results to cell size. The method for setting up the destination should be reported. The model tester should also report if the vehicle exhibits acceleration/deceleration during the movement. The tester may also show results in relation to different time intervals adopted for the estimation of flows, people densities and walking speeds. |

| **T.5** | **Vehicle speed reduction in reduced visibility conditions** |
| --- | --- |
| Objective | Assess consistency between implemented relationship between reduced speed due to smoke and hand-calculated results. |
| Geometry | A road with a single carriageway considering movement on a single lane for a total length of 1000 m (see Figure A.11). The road type should correspond to a speed limit equal to 70 km/h.  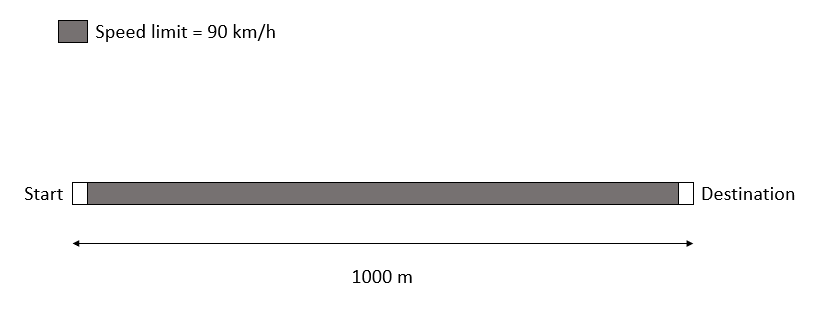  *Figure A.11. Geometry of scenario T.5.* |
| Scenario(s) | One vehicle with an assigned free flow speed corresponding to the speed limit (70 km/h) moving along the road (from start to destination), with a given set visibility value. Repeat the test varying the initial density of vehicle on the road (e.g. using 5 vehicle density levels ranging from 1 veh/km/lane to the vehicle density corresponding to a congested scenario) and five visibility values (no smoke, and four different levels of visibility, e.g. visibility corresponding to an optical density per m of 0.05 m^-1^, 0.10 m^-1^, 0.15 m^-1^and 0.20 m^-1^). While running the test case, the user should turn off any non-relevant models, except the traffic simulation model. |
| Expected result | The vehicle should cover the distance of the road in the expected time (to be calculated in accordance with the modelling assumptions adopted) |
| Test method | The test method is a quantitative verification of model results, i.e. the difference between the expected result and the simulation results. |
| User’s actions | The effectiveness of this test can be improved by setting additional prescriptions in relation to the type of model under consideration. For example, in the case of models that use a network approach, results may be dependent on the configuration of the network/grid adopted. For grid-based models, considerations should also be made by the tester on the necessity of performing this test with different configurations (e.g. simulating the default cell size and a set of both reduced and increased cell sizes) in order to test the sensitivity of the results to cell size. The method for setting up the destination should be reported. The model tester should also report if the vehicle exhibits acceleration/deceleration during the movement. |

| **T.6** | **Flow at destination** |
| --- | --- |
| Objective | Assess consistency between maximum flow rates at destination and model representation. |
| Geometry | A road with a single carriageway considering movement on a single lane for a total length of 1000 m (see Figure A.12). The road type should correspond to a speed limit equal to 90 km/h.  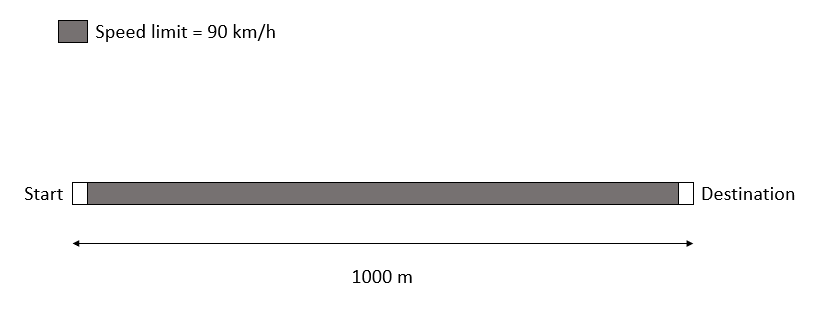  *Figure A.12. Geometry of scenario T.6.* |
| Scenario(s) | Calculate 5 vehicle density levels ranging from 1 veh/km/lane to the vehicle density corresponding to a congested scenario (Density 1=D1=1 veh/km/lane, D2, D3, D4 and D5, where D5=density leading to stopped vehicles on the road segment). The vehicles are uniformly distributed in the entire road segment. They have an initial free flow speed equal to the speed limit.  Step 1: Assign a number of vehicles corresponding to the D3 vehicle density on the road segment to move to the right towards the destination of the road segment.  Step 2: Step one is repeated with D1, D2, D4 and D5. While running the test case, the user should turn off all the non-relevant modelling layers, except the traffic simulation model.  While running the test case, the user should turn off any non-relevant models, except the traffic simulation model. |
| Expected result | The flow rate at the destination over the entire period should not exceed a pre-defined maximum threshold. |
| Test method | The test method is a quantitative evaluation of model results, i.e. the comparison between the results produced by the model and the maximum flow rate. |
| User’s actions | The effectiveness of this test can be improved by setting additional prescriptions in relation to the type of model under consideration. For example, in the case of models that use a network approach, results may be dependent on the configuration of the network/grid adopted. For grid-based models, considerations should also be made by the tester on the necessity of performing this test with different configurations (e.g. simulating the default cell size and a set of both reduced and increased cell sizes) in order to test the sensitivity of the results to cell size. The method for setting up the destination should be reported. If the model represents flows as an emergent property, the maximum flow rate for the test should be defined by the tester in relation to the underlying assumptions used during the development of the model. The model tester should document the assumptions adopted in the representation of the flows (emergent flow or user-defined). |

| **T.7** | **Group evacuation** |
| --- | --- |
| Objective | Assess consistency between the conceptual representation of group evacuation of vehicles leaving the same household and the modelled representation of group evacuation. |
| Geometry | A road with a single carriageway considering movement on a single lane with a starting point off-centre leading to two destinations for a total length of either 1000 m or 2000 m (see Figure A.13). The road type should correspond to a speed limit equal to 90 km/h.  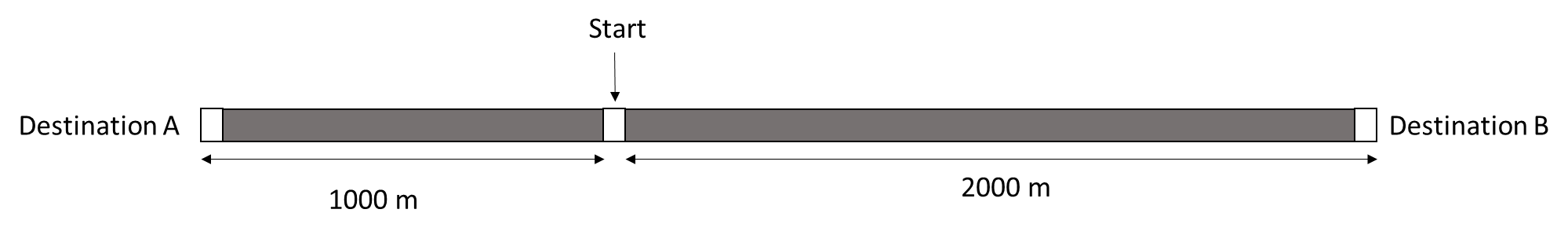  *Figure A.13. Geometry of scenario T.7.* |
| Scenario(s) | More than one vehicle leaves the household with an assigned free flow speed corresponding to the speed limit (90 km/h) moving along the road (from start to Destination B). Two sub-cases are conducted, one without any initial density on the road and one with an initial density of vehicles corresponding to 50 % of the capacity of the road. The vehicles leave the start location at the same time. While running the test case, the user should turn off any non-relevant models, except the traffic simulation model. |
| Expected result | The vehicles should take the same route to the destination and cover the distance of the road in approximately the same expected time (to be calculated in accordance with the modelling assumptions adopted) |
| Test method | The test method is a quantitative verification of model results, i.e. the difference between the expected result and the simulation results. |
| User’s actions | The effectiveness of this test can be improved by setting additional prescriptions in relation to the type of model under consideration. For example, in the case of models that use a network approach, results may be dependent on the configuration of the network/grid adopted. For grid-based models, considerations should also be made by the tester on the necessity of performing this test with different configurations (e.g. simulating the default cell size and a set of both reduced and increased cell sizes) in order to test the sensitivity of the results to cell size. The method for setting up the destination should be reported. The model tester should also report if the vehicle exhibits acceleration/deceleration during the movement. |

| **T.8** | **Lane changing/overtaking** |
| --- | --- |
| Objective | Assess consistency between conceptual vehicle capacity to overtake on a single road type and modelled representation of overtaking. |
| Geometry | A road with a single carriageway (two lanes per direction of movement for a total of four lanes) considering movement for a total length of 1000 m (see Figure A.14). The road type should correspond to a speed limit equal to 90 km/h.  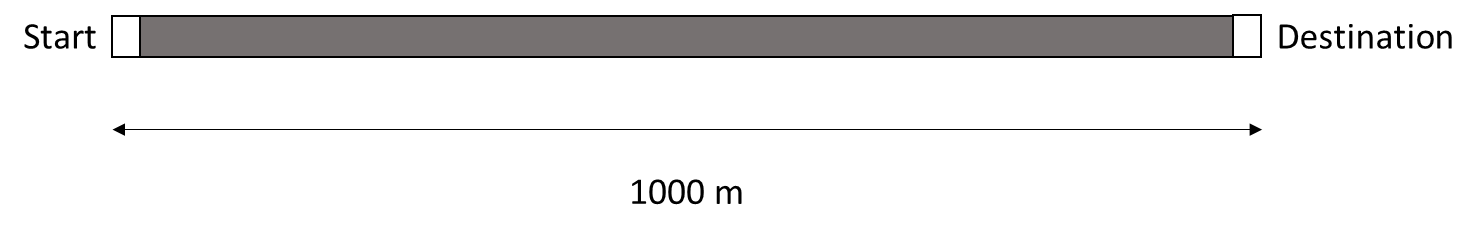  *Figure A.14. Geometry of scenario T.7.* |
| Scenario(s) | One vehicle starts with an assigned movement speed that is lower than the free flow speed moving along the road (from start to destination). Another vehicle is injected right after the first vehicle with an assigned free flow speed corresponding to the speed limit (90 km/h) moving along the road (from start to destination). While running the test case, the user should turn off any non-relevant models, except the traffic simulation model. |
| Expected result | The faster vehicle should overtake the slowest vehicle and cover the distance of the road in the expected time (to be calculated in accordance with the modelling assumptions adopted) |
| Test method | The test method is a quantitative verification of model results, i.e. the difference between the expected result and the simulation results. |
| User’s actions | The effectiveness of this test can be improved by setting additional prescriptions in relation to the type of model under consideration. For example, in the case of models that use a network approach, results may be dependent on the configuration of the network/grid adopted. For grid-based models, considerations should also be made by the tester on the necessity of performing this test with different configurations (e.g. simulating the default cell size and a set of both reduced and increased cell sizes) in order to test the sensitivity of the results to cell size. The method for setting up the destination should be reported. The model tester should also report if the vehicle exhibits acceleration/deceleration during the movement. |

| **T.9** | **Acceleration/deceleration** |
| --- | --- |
| Objective | Assess consistency between acceleration and deceleration for speed assignment of one vehicle on a single road type and model representation of acceleration and deceleration. |
| Geometry | A road with a single carriageway considering movement on a single lane for a total length of 1000 m + 1000 m (see Figure A.15) with an intersection (the user can choose the length of the intersecting road; in this example this is equal to 1000 m). The road type should correspond to a speed limit equal to 90 km/h.  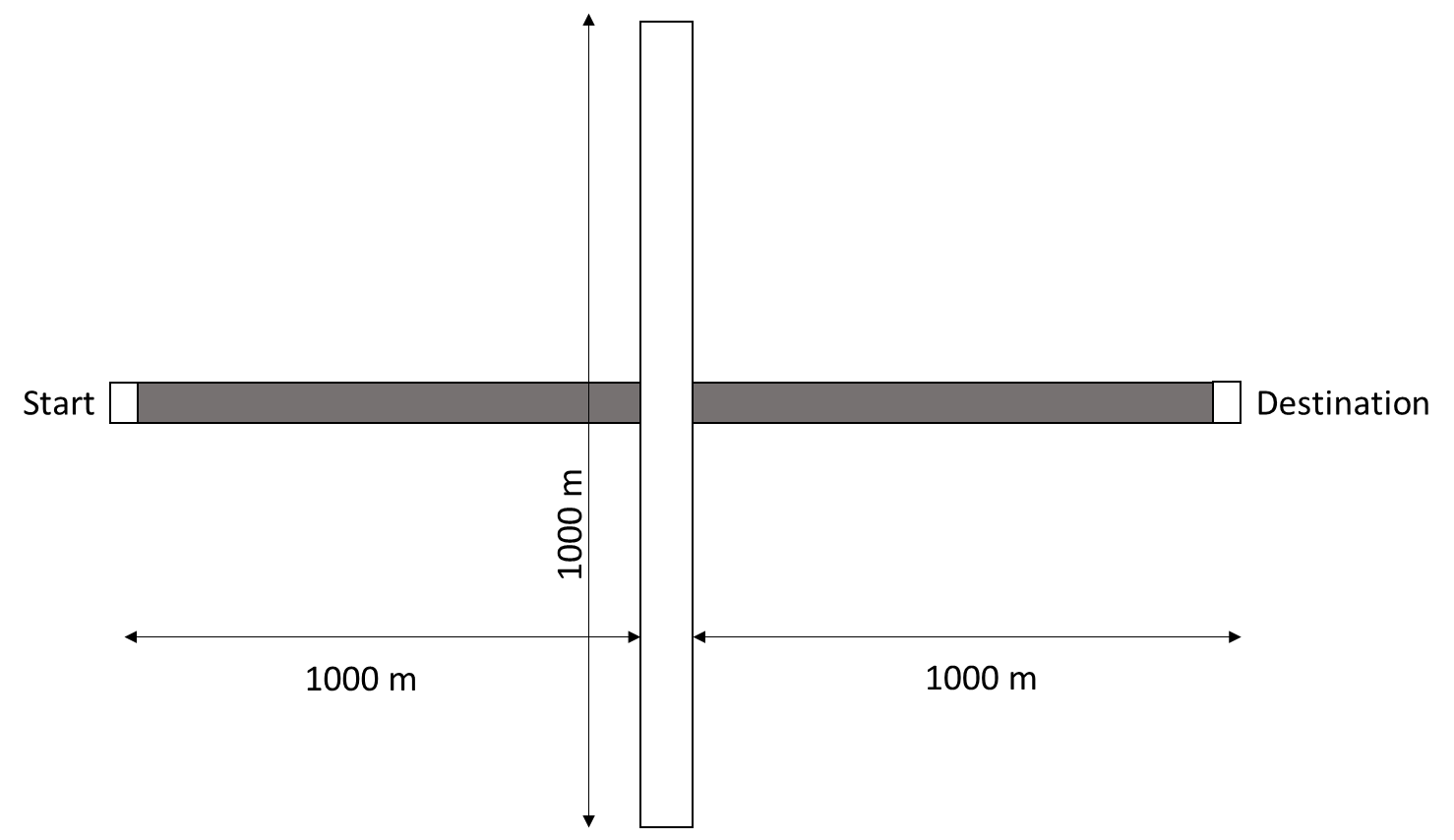  *Figure A.15. Geometry of scenario T.9.* |
| Scenario(s) | One vehicle with an assigned free flow speed corresponding to the speed limit (90 km/h) moving along the road (from start to destination) stopping at the intersection. Repeat the test removing the intersection from the road. While running the test case, the user should turn off any non-relevant models, except the traffic simulation model. |
| Expected result | The vehicle should cover the distance of the road in the expected time for both tests (to be calculated in accordance with the modelling assumptions adopted) |
| Test method | The test method is a quantitative verification of model results, i.e. the difference between the expected result and the simulation results. |
| User’s actions | The effectiveness of this test can be improved by setting additional prescriptions in relation to the type of model under consideration. For example, in the case of models that use a network approach, results may be dependent on the configuration of the network/grid adopted. For grid-based models, considerations should also be made by the tester on the necessity of performing this test with different configurations (e.g. simulating the default cell size and a set of both reduced and increased cell sizes) in order to test the sensitivity of the results to cell size. The method for setting up the destination should be reported. |

| **T.10** | **Road crash** |
| --- | --- |
| Objective | Assess the impact of road crash on traffic flow by checking consistency between the simulated evacuation time for one vehicle with a speed assignment on a single road type and model representation for road crashes. This is aimed at predicting the link response in case of crash (i.e., how the flow changes). |
| Geometry | A road with a single carriageway considering movement on a single lane for a total length of 1000 m (see Figure A.16). The road type should correspond to a speed limit equal to 90 km/h.  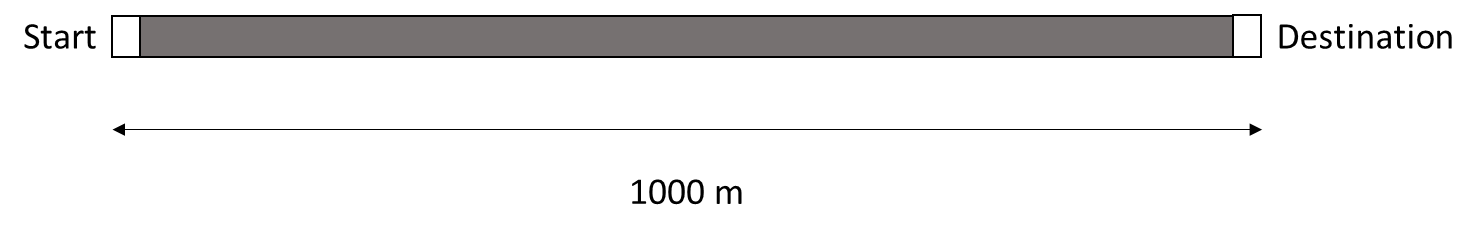  *Figure A.16. Geometry of scenario T.10.* |
| Scenario(s) | One vehicle with an assigned free flow speed corresponding to the speed limit (90 km/h) moving along the road (from start to destination). After 10 s in the simulation, an event is triggered, and the road crash is implemented in the road segment leading towards the Destination. Two sub-cases are conducted. The first changes the free-flow speed to stall speed > 0 after the implementation of the road crash, for the remaining duration of the test. The second changes the free-flow speed to stall speed equal to 0 for the remaining duration of the test. A time limitation is needed to prevent infinite simulation time. While running the test case, the user should turn off any non-relevant models, except the traffic simulation model. |
| Expected result | The vehicle should cover the distance of the road in the expected time (to be calculated in accordance with the modelling assumptions adopted) |
| Test method | The test method is a quantitative verification of model results, i.e. the difference between the expected result and the simulation results. |
| User’s actions | The effectiveness of this test can be improved by setting additional prescriptions in relation to the type of model under consideration. For example, in the case of models that use a network approach, results may be dependent on the configuration of the network/grid adopted. For grid-based models, considerations should also be made by the tester on the necessity of performing this test with different configurations (e.g. simulating the default cell size and a set of both reduced and increased cell sizes) in order to test the sensitivity of the results to cell size. The method for setting up the destination should be reported. The model tester should also report if the vehicle exhibits acceleration/deceleration during the movement. |

| **T.11** | **Intersection** |
| --- | --- |
| Objective | Assess consistency between speed assignment of one vehicle on multiple road segments and model representation for unsignalised intersections. |
| Geometry | A road with a single carriageway considering movement on a single lane for a total length of 1000 m + 1000 m (see Figure A.17) with an unsignalised intersection (the user can choose the length of the intersecting road, in this example this is equal to 1000 m). The road type before and after the intersection should correspond to a speed limit equal to 90 km/h.  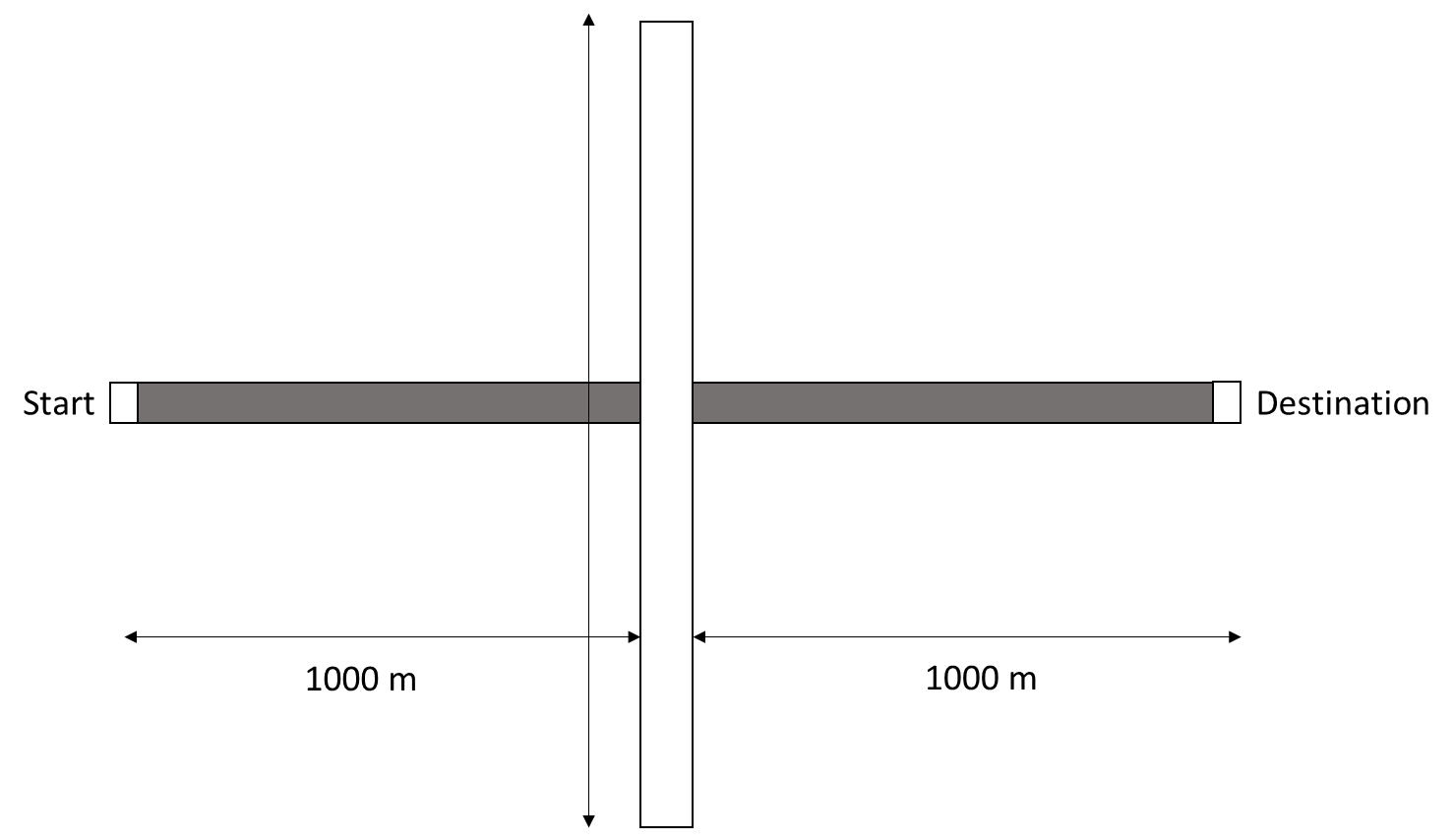  *Figure A.17. Geometry of scenario T.11.* |
| Scenario(s) | One vehicle with an assigned free flow speed corresponding to the speed limit (90 km/h) moving along the road (from start to destination). While running the test case, the user should turn off any non-relevant models, except the traffic simulation model. |
| Expected result | The vehicle should cover the distance of the road in the expected time (to be calculated in accordance with the modelling assumptions adopted) |
| Test method | The test method is a quantitative verification of model results, i.e. the difference between the expected result and the simulation results. |
| User’s actions | The effectiveness of this test can be improved by setting additional prescriptions in relation to the type of model under consideration. For example, in the case of models that use a network approach, results may be dependent on the configuration of the network/grid adopted. For grid-based models, considerations should also be made by the tester on the necessity of performing this test with different configurations (e.g. simulating the default cell size and a set of both reduced and increased cell sizes) in order to test the sensitivity of the results to cell size. The method for setting up the destination should be reported. The model tester should also report if the vehicle exhibits acceleration/deceleration during the movement. |

| **T.12** | **Forced Destination** |
| --- | --- |
| Objective | Assess consistency between the conceptual implementation of a forced destination and the model representation of a forced destination. |
| Geometry | A road with a single carriageway considering movement on a single lane with a starting point off-centre leading to two destinations for a total length of either 1000 m or 2000 m (see Figure A.18). The road type should correspond to a speed limit equal to 90 km/h.  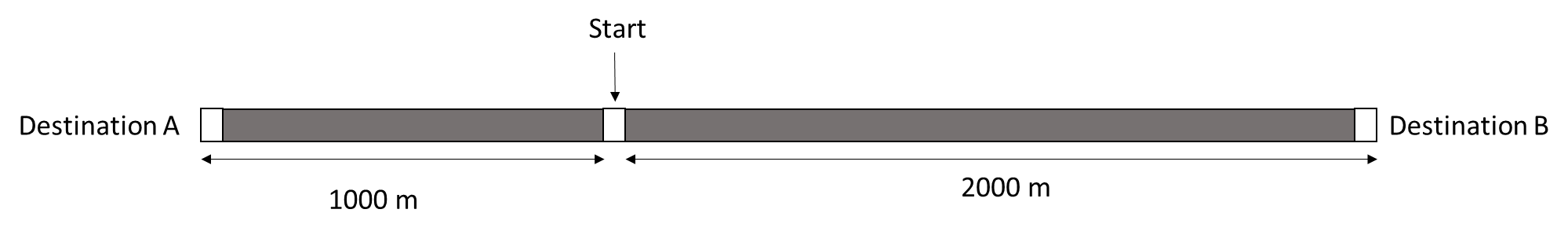  *Figure A.18. Geometry of scenario T.12.* |
| Scenario(s) | One vehicle with an assigned free flow speed corresponding to the speed limit (90 km/h) moving along the road (from start to destination). The vehicle is forced to go towards Destination B through the implementation of a forced destination. If forced destinations cannot be implemented, the vehicle would by default drive towards Destination A since it is both the closest and fastest route. While running the test case, the user should turn off any non-relevant models, except the traffic simulation model. |
| Expected result | The vehicle should cover the distance and drive to Destination B. |
| Test method | The test method is a quantitative verification of model results, i.e. the difference between the expected result and the simulation results. |
| User’s actions | The effectiveness of this test can be improved by setting additional prescriptions in relation to the type of model under consideration. For example, in the case of models that use a network approach, results may be dependent on the configuration of the network/grid adopted. The method for setting up the destination should be reported. |

| **T.13** | **Destination choice in traffic** |
| --- | --- |
| Objective | Assess consistency between the conceptual implementation of destination choice and model representation of destination choice. |
| Geometry | A road with a single carriageway considering movement on a single lane with a starting point off-centre leading to two destinations for a total length of either 1000 m or 2000 m (see Figure A.19). The road leading towards Destination A corresponds to a speed limit equal to 30 km/h for 1000 m. The road leading towards Destination B corresponds to a speed limit equal to 120 km/h for 2000 m.  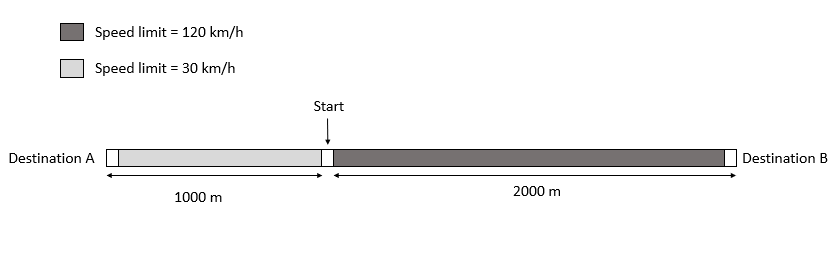  *Figure A.19. Geometry of scenario T.13.* |
| Scenario(s) | One vehicle with an assigned free flow speed corresponding to the speed limits (30 km/h and 120 km/h) moving along the road (from start to destination). Repeat the test for each destination choice method that is available (e.g. destination based on shortest route, fastest route, any other condition such as smoke that affects the selection). While running the test case, the user should turn off any non-relevant models, except the traffic simulation model. |
| Expected result | The vehicle should drive towards the correct destination that corresponds to the route choice made and cover the distance of the road in the expected time (to be calculated in accordance with the modelling assumptions adopted) |
| Test method | The test method is a quantitative verification of model results, i.e. the difference between the expected result and the simulation results. |
| User’s actions | The effectiveness of this test can be improved by setting additional prescriptions in relation to the type of model under consideration. The method for setting up the destination should be reported. The model tester should also report if the vehicle exhibits acceleration/deceleration during the movement. |

| **T.14** | **Route choice in traffic** |
| --- | --- |
| Objective | Assess consistency between the conceptual implementation of destination choice and model representation of destination choice. |
| Geometry | A road with a single carriageway considering movement on a single lane with a starting point connecting two separate roads leading to the same destination for a total length of either 4000 m or 2000 m (see Figure A.20). The road type for the longer route should correspond to a speed limit equal to 120 km/h. The road type for the shorter route should correspond to a speed limit equal to 30 km/h.  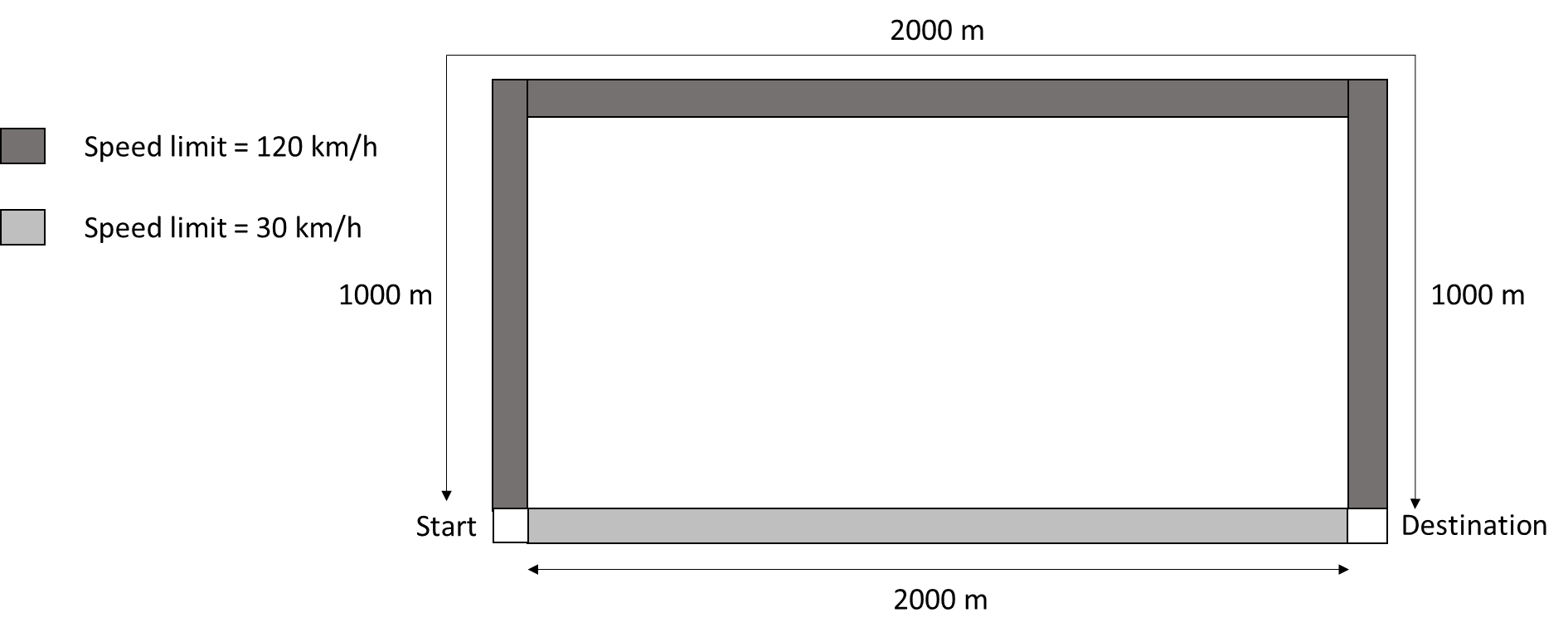  *Figure A.20. Geometry of scenario T.14.* |
| Scenario(s) | One vehicle with an assigned free flow speed corresponding to the speed limits (30 km/h and 120 km/h) moving along the road (from start to destination). Repeat the test for each destination choice method that is available (e.g. destination based on shortest route, fastest route, any other condition such as smoke that affects the selection). While running the test case, the user should turn off any non-relevant models, except the traffic simulation model. |
| Expected result | The vehicle should drive to the correct route that corresponds to the route choice made and cover the distance of the road in the expected time (to be calculated in accordance with the modelling assumptions adopted) |
| Test method | The test method is a quantitative verification of model results, i.e. the difference between the expected result and the simulation results. |
| User’s actions | The effectiveness of this test can be improved by setting additional prescriptions in relation to the type of model under consideration. The method for setting up the destination should be reported. |

| **T.15** | **Vehicle demand vs arrival distribution** |
| --- | --- |
| Objective | Assess consistency between implemented relationship for number of vehicles distributed in the traffic system and hand-calculated result. |
| Geometry | A road with a single carriageway considering movement on a single lane for a total length of 1000 m (see Figure A.21). The road type should correspond to a speed limit equal to 90 km/h.  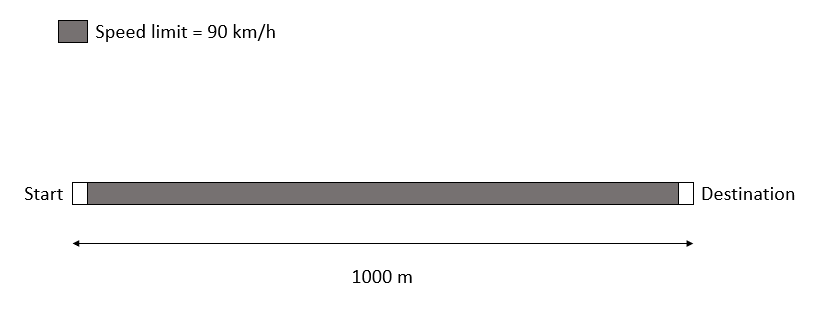  *Figure A.21. Geometry of scenario T.15.* |
| Scenario(s) | Implement given numbers of vehicles (2, 50, and 100) with an assigned free flow speed corresponding to the speed limit (90 km/h) moving along the road (from start to destination). While running the test case, the user should turn off any non-relevant models, except the traffic simulation model. |
| Expected result | The number of vehicles reaching the destination should correspond to the implemented number of vehicles assigned to the traffic system (to be calculated in accordance with the modelling assumptions adopted). |
| Test method | The test method is a quantitative verification of model results, i.e. the difference between the expected result and the simulation results. |
| User’s actions | The effectiveness of this test can be improved by setting additional prescriptions in relation to the type of model under consideration. For example, in the case of models that use a network approach, results may be dependent on the configuration of the network/grid adopted. For grid-based models, considerations should also be made by the tester on the necessity of performing this test with different configurations (e.g. simulating the default cell size and a set of both reduced and increased cell sizes) in order to test the sensitivity of the results to cell size. The method for setting up the destination should be reported. |

| **WT.1** | **Route loss** |
| --- | --- |
| Objective | Assess consistency between the conceptual implementation of route loss (e.g., generated by the fire) and the model representation of route loss. |
| Geometry | A road with a single carriageway considering movement on a single lane with an intersection with two roads leading to the same destination for a total length of either 1000 m + 4000 m, or 1000 m + 2000 m (see Figure A.22). The road type should correspond to a speed limit equal to 90 km/h.  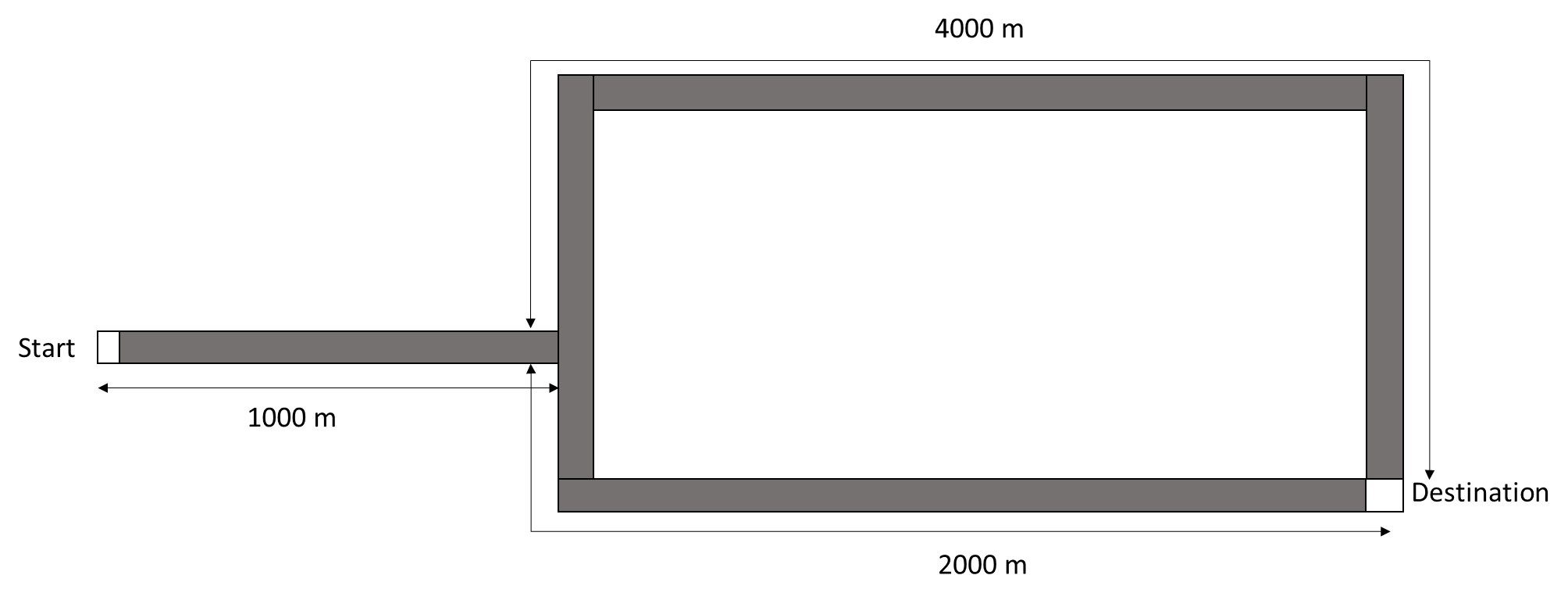  *Figure A.22. Geometry of scenario WT.1.* |
| Scenario(s) | One vehicle with an assigned free flow speed corresponding to the speed limit (90 km/h) moving along the road (from start to destination). After 10 s in the simulation (before the vehicle leaves the first segment), an event is triggered, and the 2000 m road before the Destination is closed. The vehicle would by default drive to the Destination via the shorter route since it is both a closer and faster route. While running the test case, the user should turn off any non-relevant models, except the traffic simulation model. |
| Expected result | The vehicle should change its route after the event is implemented to the longer route, cover the distance of the road in the expected time (to be calculated in accordance with the modelling assumptions adopted, e.g. the time of the longer route) |
| Test method | The test method is a quantitative verification of model results, i.e. the difference between the expected result and the simulation results. |
| User’s actions | The effectiveness of this test can be improved by setting additional prescriptions in relation to the type of model under consideration. For example, in the case of models that use a network approach, results may be dependent on the configuration of the network/grid adopted. The method for setting up the destination should be reported. The user should also report the impact of the loss of route for the vehicles that are on the loss route on the moment when this is triggered. |

| **WT.2** | **Lane reversal** |
| --- | --- |
| Objective | Assess the impact of lane reversal (e.g. by increasing road capacity) by checking the consistency between the simulated evacuation time and the calculated one of a vehicle on a single road type with given traffic densities for uni-directional movement. |
| Geometry | A road with a single carriageway considering movement on a single lane for a total length of 1000 m (see Figure A.23). The road type should correspond to a speed limit equal to 90 km/h.  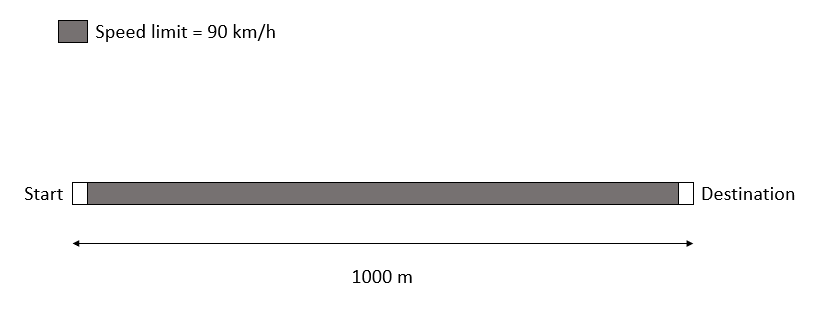  *Figure A.23. Geometry of scenario WT.2.* |
| Scenario(s) | One vehicle with an assigned free flow speed corresponding to the speed limit (90 km/h) moving along the road (from start to destination). After 30 s in the simulation, an event is triggered and lane reversal is implemented in the entire road segment. Repeat the test varying the initial density of vehicle on the road (e.g., using 5 vehicle density levels linearly from 1 veh/km/lane to the vehicle density corresponding to the vehicle being stopped considering the portion of the road before the implementation of the lane reversal). While running the test case, the user should turn off any non-relevant models, except the traffic simulation model. |
| Expected result | The vehicle should cover the distance of the road in the expected time (to be calculated in accordance with the modelling assumptions adopted) |
| Test method | The test method is a quantitative verification of model results, i.e. the difference between the expected result and the simulation results. |
| User’s actions | The effectiveness of this test can be improved by setting additional prescriptions in relation to the type of model under consideration. For example, in the case of models that use a network approach, results may be dependent on the configuration of the network/grid adopted. For grid-based models, considerations should also be made by the tester on the necessity of performing this test with different configurations (e.g. simulating the default cell size and a set of both reduced and increased cell sizes) in order to test the sensitivity of the results to cell size. The method for setting up the destination should be reported. The model tester should also report if the vehicle exhibits acceleration/deceleration during the movement. |

| **WT.3** | **Loss of exit or shelter** |
| --- | --- |
| Objective | Assess consistency between the conceptual implementation of loss of exit/shelter and the model representation of loss of exit/shelter. |
| Geometry | A road with a single carriageway considering movement on a single lane with an intersection leading to two different destinations for a total length of either 1000 m + 1000 m, or 1000 m + 2000 m (see Figure A.24). The road type should correspond to a speed limit equal to 90 km/h.  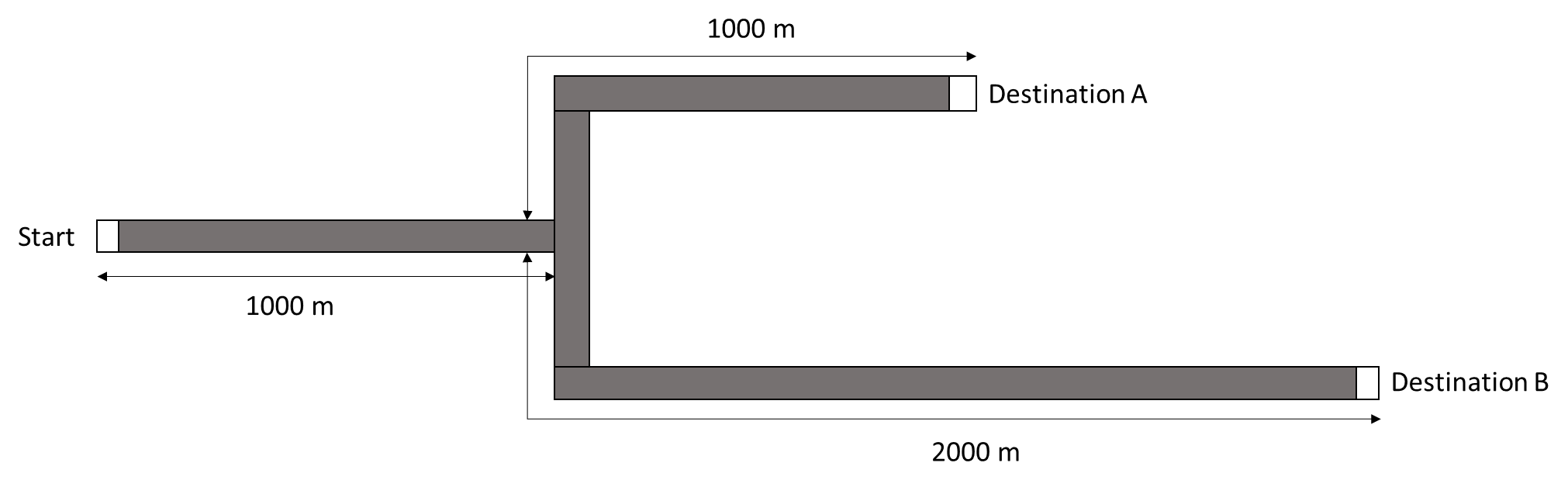  *Figure A.24. Geometry of scenario WT.3.* |
| Scenario(s) | One vehicle with an assigned free flow speed corresponding to the speed limit (90 km/h) moving along the road (from start to destination). After 30 s in the simulation, an event is triggered, and Destination A is closed. The vehicle would by default drive towards Destination A since it is both a closer and faster route. While running the test case, the user should turn off any non-relevant models, except the traffic simulation model. |
| Expected result | The vehicle should change its destination after the event is implemented, cover the distance and drive to Destination B. |
| Test method | The test method is a quantitative verification of model results, i.e. the difference between the expected result and the simulation results. |
| User’s actions | The effectiveness of this test can be improved by setting additional prescriptions in relation to the type of model under consideration. The method for setting up the destination should be reported. The user should also report the impact of the loss of exit/shelters on the vehicles that are closely approaching it. |

| **WT.4** | **Refuge capacity** |
| --- | --- |
| Objective | Ensure the impact of refuge reaching its full capacity and the re-direction of traffic to the next refuge is correctly implemented. |
| Geometry | A road with a single carriageway considering movement on a single lane with an intersection leading to two different destinations for a total length of either 1000 m + 1000 m, or 1000m + 2000 m (see Figure A.25). The road type should correspond to a speed limit equal to 90 km/h.  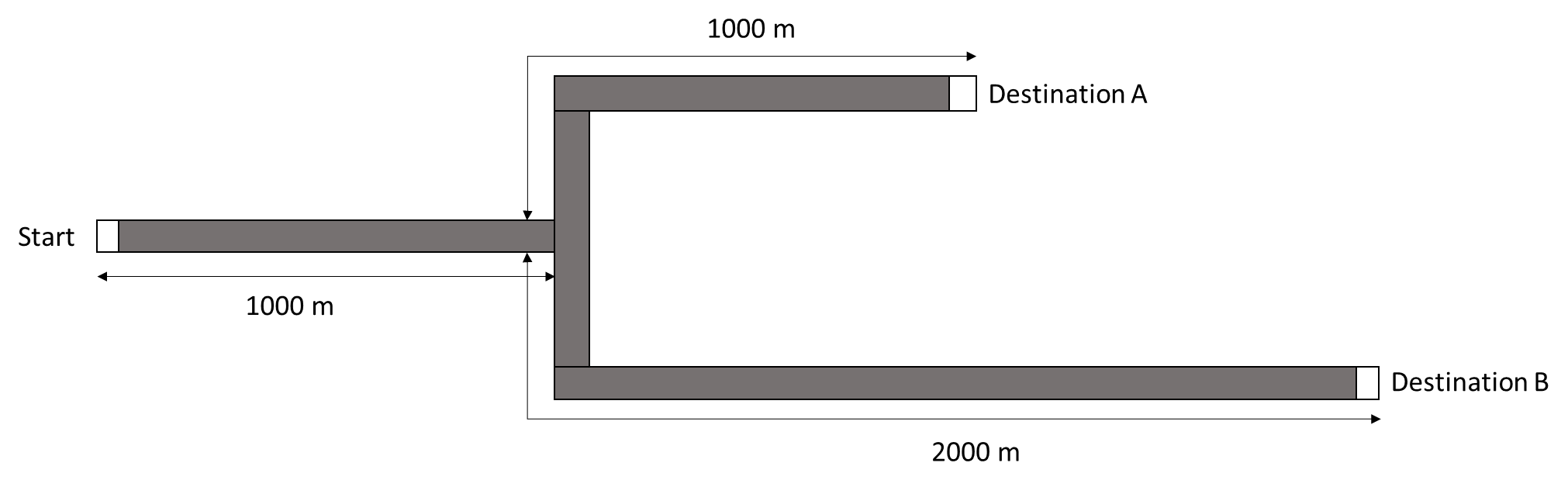  *Figure A.25. Geometry of scenario WT.4.* |
| Scenario(s) | Two vehicles with an assigned free flow speed corresponding to the speed limit (90 km/h) moving along the road (from start to destination). Both Destination A and B are refuges with capacity as emergency shelter for one person. The vehicles would by default drive towards Destination A since it is both a closer and faster route. While running the test case, the user should turn off any non-relevant models, except the traffic simulation model. |
| Expected result | The vehicles should cover the distance and drive to Destination A. When the first vehicle has reached the refuge and filled up its capacity, the second vehicle will change route and drive to Destination B. |
| Test method | The test method is a quantitative verification of model results, i.e. the difference between the expected result and the simulation results. |
| User’s actions | The effectiveness of this test can be improved by setting additional prescriptions in relation to the type of model under consideration. For example, in the case of models that use a network approach, results may be dependent on the configuration of the network/grid adopted. For grid-based models, considerations should also be made by the tester on the necessity of performing this test with different configurations (e.g. simulating the default cell size and a set of both reduced and increased cell sizes) in order to test the sensitivity of the results to cell size. The method for setting up the destination should be reported. |
